# Gene expression meta-analysis reveals immune response convergence on the IFNγ-STAT1-IRF1 axis and adaptive immune resistance mechanisms in lymphoma

**DOI:** 10.1186/s13073-015-0218-3

**Published:** 2015-09-11

**Authors:** Matthew A. Care, David R. Westhead, Reuben M. Tooze

**Affiliations:** Section of Experimental Haematology, Wellcome Trust Brenner Building, Leeds Institute of Cancer and Pathology, University of Leeds, Leeds, LS9 7TF UK; Bioinformatics Group, School of Molecular and Cellular Biology, University of Leeds, Leeds, UK

## Abstract

**Background:**

Cancers adapt to immune-surveillance through evasion. Immune responses against carcinoma and melanoma converge on cytotoxic effectors and IFNγ-STAT1-IRF1 signalling. Local IFN-driven immune checkpoint expression can mediate feedback inhibition and adaptive immune resistance. Whether such coupled immune polarization and adaptive resistance is generalisable to lymphoid malignancies is incompletely defined. The host response in diffuse large B-cell lymphoma (DLBCL), the commonest aggressive lymphoid malignancy, provides an empirical model.

**Methods:**

Using ten publicly available gene expression data sets encompassing 2030 cases we explore the nature of host response in DLBCL. Starting from the “cell of origin” paradigm for DLBCL classification, we use the consistency of differential expression to define polarized patterns of immune response genes in DLBCL, and derive a linear classifier of immune response gene expression. We validate and extend the results in an approach independent of “cell of origin” classification based on gene expression correlations across all data sets.

**Results:**

T-cell and cytotoxic gene expression with polarization along the IFNγ-STAT1-IRF1 axis provides a defining feature of the immune response in DLBCL. This response is associated with improved outcome, particularly in the germinal centre B-cell subsets of DLBCL. Analysis of gene correlations across all data sets, independent of “cell of origin” class, demonstrates a consistent association with a hierarchy of immune-regulatory gene expression that places *IDO1*, *LAG3* and *FGL2* ahead of PD1-ligands *CD274* and *PDCD1LG2*.

**Conclusion:**

Immune responses in DLBCL converge onto the IFNγ-STAT1-IRF1 axis and link to diverse potential mediators of adaptive immune resistance identifying future therapeutic targets.

**Electronic supplementary material:**

The online version of this article (doi:10.1186/s13073-015-0218-3) contains supplementary material, which is available to authorized users.

## Background

Emergence of clinically detectable malignant disease is associated with escape from tumour immune surveillance [1]. Two principal mechanisms may operate: on the one hand the immune systems loses the ability to detect the neoplastic population through changes in antigen presentation or editing of the antigen receptor repertoire; on the other hand initially effective immune responses may be rendered ineffective through development of an immune suppressive environment [2]. In the latter scenario, local expression of immune checkpoint components can be viewed as subversion of a physiological mechanism, which acts during chronic infections to balance effective immunity with immune-mediated tissue damage [3].

In a range of cancers the density, location and functional polarization of tumour infiltrating lymphocytes are of prognostic value [4], providing evidence that the nature of immune evasion remains of importance after clinical detection. This is particularly relevant in the context of novel therapeutic strategies aimed at re-invigorating the “exhausted” anti-tumour immune response through immune checkpoint blockade [5, 6]. Gene expression analysis of bulk tumour tissue integrates expression profiles from multiple cellular sources, often allowing global assessment of the predominant vector of functional immune polarization. A paradigm has been proposed in which cancer-associated immune responses converge on a common “immunologic constant of rejection” characterized by a pattern of cytotoxic and T-cell immune responses and a dominant IFNγ-STAT1-IRF1 signalling axis [4, 7]. Linking the polarized pattern of interferon (IFN)γ-driven immune responses to the expression of immune checkpoints is the concept of “adaptive immune resistance” [5, 8]. In this model IFNγ signalling drives local feedback inhibition through the transcriptional regulation of ligands for the inhibitory receptor PD1 [5, 8]. The common association between cytotoxic responses and expression of IFN signatures and potential mediators of adaptive immune resistance has been further supported by analysis of solid tumour gene expression data from The Cancer Genome Atlas [9]. Importantly, such feedback may be mediated both at the immediate interface between tumour cell and cytotoxic lymphocyte, and by the establishment of a wider immune suppressive milieu in the tumour microenvironment.

The combination of convergent IFN-polarized immune responses [4, 7], coupled to IFN-driven adaptive immune resistance [5, 8], provides a powerful model with which to explain common pathologic associations in carcinoma and melanoma. The recent success of therapies targeting CTLA4 and PD1 immune checkpoints [10–12], combined with an extended range of other therapeutic options [6], means that evidence to support prioritization of therapeutic combinations in different tumour settings is required. Lymphoma, which comprises immune system malignancies, provides an instance in which these pathways are incompletely studied. Classical Hodgkin lymphoma is the archetype in which host response elements dominate to the point of obscuring the neoplastic B-cell clone [13], and in classical Hodgkin lymphoma PD1 pathway blockade has recently been described as a promising therapeutic approach [14]. Diffuse large B-cell lymphoma (DLBCL) is the commonest form of nodal lymphoma in the western world and represents an aggressive malignancy that frequently remains incurable. It is well established that this lymphoma type is associated with a varied extent of host response at diagnosis, which can include elements of IFN signalling [15]. Since several large data sets are publicly available [15–25], this malignancy represents an empirical human model in which to test the association between immune polarization and adaptive immune resistance mechanisms.

The “cell of origin” (COO) classification provides the dominant paradigm for our current understanding of DLBCL [24, 26]. This classification relates the gene expression profiles in DLBCL to those of germinal centre B cells (GCBs) or activated B cells (ABCs), the latter representing the initial stage of B-cell terminal differentiation to plasma cells. Although the COO classification allows the division of DLBCL based on expression of a restricted set of classifier genes into the two principal classes [24], a subset of cases show patterns of classifier gene expression that do not allow confident assignment to either GCB or ABC subsets. Such cases are referred to as “type 3” [24, 26], or “unclassified” [27, 28]. To avoid ambiguity we refer to these cases as COO-unclassified DLBCL in the following. In a parallel “consensus cluster” classification developed by Monti et al. [15], it was shown that DLBCL could be divided into three categories characterized by preferential expression of genes linked to proliferation and B-cell receptor signalling, metabolic oxidative phosphorylation, or host response. The latter included multiple elements attributable to components of the immune system and supporting stromal cell types. It was noted that a greater proportion of COO-unclassified DLBCL belonged to the host/immune response cluster, which had increased numbers of intra-tumoral T cells and macrophages and a relative decrease in neoplastic B cells [15].

We reasoned that the potential association of COO-unclassified DLBCL with intense host responses provided a starting point for a meta-analysis of immune response elements in DLBCL. In originating from a prevailing paradigm this provided a wider biological and clinical context. Furthermore, by asking whether evidence supporting a common polarized immune response could be discovered from within the construct of the COO paradigm, we sought to avoid bias that might have arisen by focusing ab initio on components of the polarized immune response or immune checkpoints. With this approach we identify a distinct signature characterised by a pattern of cytotoxic T-cell and IFNγ-polarized immune response genes as a dominant pattern across ten DLBCL data sets encompassing 2030 cases. Using components of this polarized pattern we then explore the immune context of DLBCL in a fashion independent of COO class. We demonstrate the strong association with an IFNγ-STAT1-IRF1 axis and an expression hierarchy of immune checkpoints/modulators, consistent with adaptive immune resistance as a common feature operating in DLBCL.

## Methods

### Data sets

Ten DLBCL data sets were downloaded from the Gene Expression Omnibus (GEO) [29] [GEO:GSE4475, GSE10846, GSE12195, GSE19246, GSE22470, GSE22895, GSE31312, GSE32918, GSE34171 and elsewhere [15–25]. GSE10846 was split according to treatment groups (CHOP [cyclophosphamide, doxorubicin hydrochloride (hydroxydaunomycin), vincristine sulfate (Oncovin), prednisone]/R-CHOP [rituximab-CHOP]), which were treated independently for analysis, thus giving a total of 11 data sets.

### Normalisation and re-annotation of data

For each data set the probes were re-annotated with the latest version of HUGO Gene Nomenclature Committee (HGNC)-approved symbols [30]. The complete HGNC list was downloaded (on 1 October 2014). Each probe was re-annotated to the latest approved symbol if an unambiguous mapping (i.e. single symbol mapping to approved symbol) could be determined, else the original gene name was maintained.

Each data set was quantile normalised using the R Limma package [31]. The probes for each gene were merged by taking the median value for probe sets with a Pearson correlation ≥0.2 and the maximum value for those with a correlation <0.2 [15].

### COO classifications

We used the COO classifications assigned by the DLBCL automatic classifier (DAC) classifier in our previous work [32].

### Meta-profile generation

See Additional file 1 for an outline of meta-profile generation using COO classification.

For each of the 11 data sets a linear model was fitted to the gene expression data using the R Limma package. Differentially expressed genes between the three classes were gauged using the Limma empirical Bayes statistic module, adjusting for multiple testing using Benjamini and Hochberg correction.

The absolute fold changes for all genes per data set were normalised between 0 and 1. The results were merged across data sets retaining only genes with an adjusted *p* value (false discovery rate, FDR < 0.05. A meta-profile was created for each contrast (e.g. upABC_GCB) by retaining all genes differentially expressed in six or more data sets. These were then used to draw Wordles [33] with each gene’s score set to (NumDataSets^3^) × NormalisedFoldChange.

### Signature enrichment analysis

A data set of 14,104 gene signatures was created by merging signatures downloaded from SignatureDB [34], MSigDB v.4 (MSigDB C1--C7) [35], Gene Signature Database v.4 (GeneSigDB) [36] and the work of Monti et al. [15] and others [37–40]. Enrichment of meta-profiles against signatures was assessed using a hypergeometric test, where the draw is the meta-profile genes, the successes are the signature genes and the population is the genes present on the platform.

### Gene ontology analysis

Meta-profile gene lists were assessed for gene ontology (GO) enrichment using the Cytoscape BiNGO tool [41]. GO and annotation files were downloaded from [42] (13 June 2014). The background reference was set to a non-redundant list of the genes present in the 11 data sets. The FDR rate (Benjamini and Hochberg) was set to ≤0.1.

### Signature enrichment visualisation

See Additional file 2 for an outline of the process for integrating and visualizing analysis of gene signature and ontology enrichments.

The results from gene signature and gene ontology enrichment were used to create heatmap visualisations. For each meta-profile the top 100 most enriched signatures and 100 most enriched GO terms were used to construct a matrix of signatures against genes. This is a binary matrix with 1 s depicting an assigned signature/GO annotation. Using Python a row-wise (gene correlation) and column-wise (signature correlation) phi coefficient was calculated. These were then hierarchical clustered using GENE-E [43] with complete linkage.

### Focus gene analysis

See Additional file 3 for an outline of the focus gene approach.

Per data set the genes were ordered by their variance across the patient samples, and the top 80 % were used to calculate Spearman’s rank correlations per row using the Python scipy.stats package. The resultant *p* value and correlation matrices were merged across the 11 data sets by taking the median values (across the sets in which the gene was contained), giving a final matrix of length 20,121. For a given focus gene the median rho and *p* values were reported along with a breakdown of the correlations and relative expression levels across the data sets (Additional file 4). For select focus genes a correlated gene set was created by taking all genes with a *p* > 0.45 present in six or more data sets. These correlated gene sets were then used for signature enrichment analysis and visualisation.

### Survival analysis

The Survival library for R was used to analyse right-censored survival data. Overall survival was estimated using the Kaplan-Meier method, modelled with Cox Proportional Hazards technique. Survival analysis was restricted to data sets of cases treated with the currently standard immunochemotherapy regimen R-CHOP.

## Results

### Shared meta-profiles for COO-unclassified and COO-classified DLBCL

**Fig. 1:**
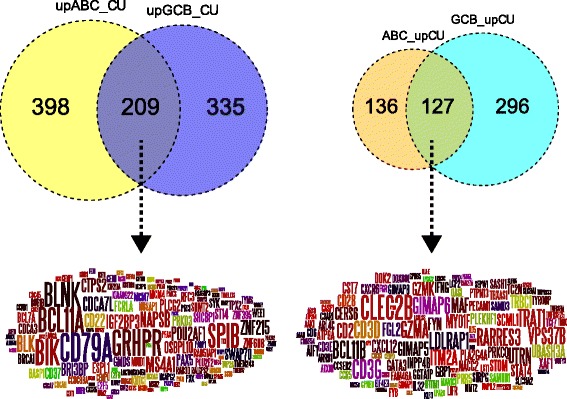
Consistent gene expression differences separate COO-unclassified DLBCL from either principal COO class. The overlap of genes consistently associated with either COO-classified DLBCL (*left* Venn diagram and Wordle) or COO-unclassified DLBC (*right* Venn diagram and Wordle) are shown. *Left*: the Venn diagram shows genes up-regulated in ABC (*yellow*) or GCB (*blue*) relative to COO-unclassified. *Right*: the Venn diagram shows genes up-regulated in COO-unclassified relative to ABC-DLBCL (*brown*) or GCB-DLBCL (*turquoise*). For the Wordles, word size is given by differential expression (*between contrasts*) to the power of median-fold change

Given the importance of the COO paradigm to both the biological and clinical assessment of DLBCL, we anchored our initial analysis on this classification. We previously developed a COO classifier implementation that allows the robust classification of multiple DLBCL data sets [32], which is currently in clinical usage in the context of a phase 3 clinical trial [44]. Applying this to the 11 largest publicly available DLBCL data sets (GSE10846 was split according to treatment into CHOP and R-CHOP components), encompassing 2030 cases [15–25], provided a resource for gene expression meta-analysis. To determine genes consistently linked to COO class we used both the consistency of differential expression between data sets as well as absolute level of differential expression to identify and rank genes associated with each class. We restricted the gene lists by applying a threshold of differential expression in 6 out of 11 data sets; we refer to these as meta-profiles. To explore the relationship of COO-unclassified DLBCL to each of the principal COO classes, we employed sequential pairwise comparisons (Additional file 1). From the initial comparison, we identified 127 genes associated with COO-unclassified DLBCL relative to both ABC- and GCB-DLBCL, while 209 genes were associated with both COO classes relative to COO-unclassified DLBCL (Additional file 5; Fig. 1). The extent of overlap was highly significant (*p* = 1.32E-157 and *p* = 2.09E-200 for genes associated with COO-unclassified DLBCL or COO class, respectively). We subsequently refer to these sets of overlapping genes as COO-unclassified and COO-classified meta-profiles, respectively.

### COO-unclassified DLBCL is enriched for features of a polarized immune response

To assess underlying biology in the COO-classified and COO-unclassified meta-profiles we developed an approach for integrated analysis of GO and gene signature enrichment (Additional file 2) which applies hierarchical clustering to reciprocally assess the relationships of enriched ontology and signature terms and associated genes contributing to enrichments (Additional file 6). The results are displayed as heatmaps of the hierarchically clustered correlations.

**Fig. 2:**
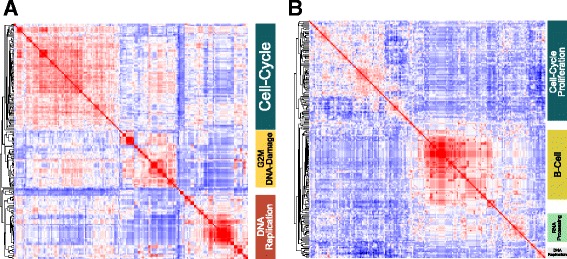
Integrated gene signature and ontology enrichment analysis demonstrates association of the COO-classified meta-profile with cell proliferation and B-cell signatures. **a** The top gene signature and ontology terms enriched in the COO-classified meta-profile, clustered according to the correlation of signatures given their gene membership. **b** The corresponding clustering of genes contributing to signature and ontology term enrichments for the COO-classified meta-profile, clustered according to correlation of genes given their signature membership. To the right general categories corresponding to major correlation clusters are illustrated. Corresponding high resolution versions are available in Additional files 7 and 8

In the COO-classified meta-profile a striking representation of genes linked to cell proliferation resulted in multiple distinct clusters of enriched terms reflecting a wide range of processes associated with cell proliferation (Fig. 2a; Additional file 7). In addition to this, distinct enrichment of signatures of the B-cell lineage was evident. From the gene perspective this was reflected in one main branch associated with cell cycle and cell proliferation, and the second including two principal subclusters associated on the one hand with RNA binding and processing, and on the other with core B-cell-associated genes (Fig. 2b; Additional file 8).

**Fig. 3:**
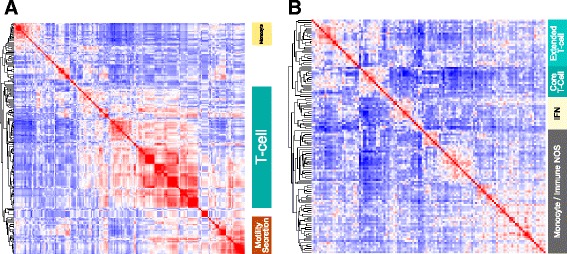
Integrated gene signature and ontology enrichment analysis demonstrates association of the COO-unclassified meta-profile with polarized immune response. **a** The top gene signature and ontology terms enriched in the COO-unclassified meta-profile, clustered according to the correlation of signatures given their gene membership. **b** The corresponding clustering of genes contributing to signature and ontology term enrichments for the COO-unclassified meta-profile, clustered according to correlation of genes given their signature membership. To the right general terms corresponding to major correlation clusters are illustrated (*NOS* not otherwise specified). Corresponding high resolution versions are available in Additional files 9 and 10

In contrast the COO-unclassified meta-profile was linked to terms related to T-cell populations, T-cell receptor signalling and T-cell activation. While the second principal branch of ontology/signature terms was linked to additional more diverse immune response elements (Fig. 3a; Additional file 9). Hierarchical clustering from the gene perspective (Fig. 3b; Additional file 10) generated a principal branch related to T cells composed of a cluster of genes representing core elements of the T-cell state (*CD2*, *CD3D*, *CD3E*, *CD3G*, *CD28* and *TRBC1*) and another cluster of genes with T-cell associations, including *BCL11B*, *GZMA*, *GZMK*, *MAF* and *STAT4*. The second principal branch of the hierarchical tree included genes derived from monocytes and other immune/host response signatures. This also included a subcluster comprising *IFNG*, and interferon responsive genes *GBP1* and *IFITM1*, as well as the chemokine receptors *CCR5*, *CXCR3* and *CXCR6*, which are linked to Th1 polarized T-cell populations [45, 46]. We therefore conclude that COO-unclassified DLBCL is generally distinguished from COO-classified DLBCL by a predominant T-cell immune response with skewing toward *IFNG* gene expression. Furthermore the paucity of both proliferation and B-cell gene expression is indicative of a relatively low representation of neoplastic B cells.

### A cytotoxic and interferon polarized immune response as an independent molecular feature of DLBCL

**Fig. 4:**
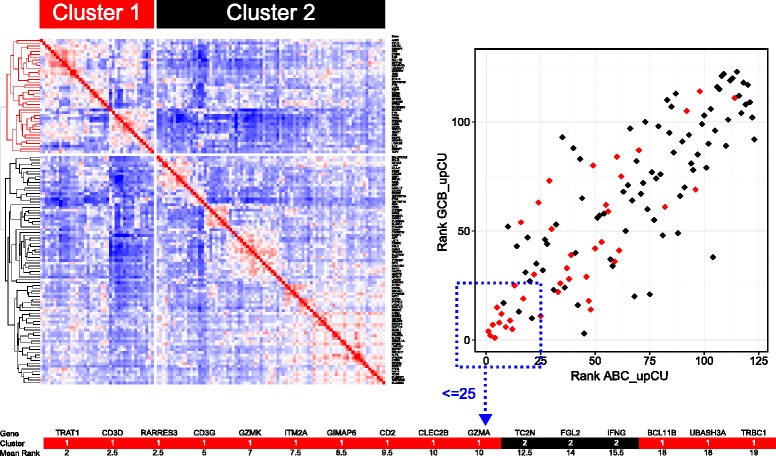
Genes most consistently associated with COO-unclassified DLBCL are related to a polarized immune response. The two principal branches of the gene-centred hierarchical clustering tree of the COO-unclassified meta-profile are illustrated on the *left*. Colour-coding identifies: *red* cluster 1, corresponding to the T-cell cluster; *black* cluster 2, IFN and monocyte/immune *NOS* (not otherwise specified). On the right the relative rank of differentially expressed genes contributing to the COO-unclassified meta-profile is plotted using a differential expression ranking, derived from the number of data sets with differential expression to the power of normalized median fold change; the x-axis indicates differential expression rank in the comparison COO-unclassified versus ABC-DLBCL; the y-axis indicates differential expression rank in the comparison COO-unclassified versus GCB-DLBCL. Cluster membership is superimposed on the scatter plot of differential expression rank according to the colour coding shown (*red* cluster 1, *black* cluster 2). The 16 genes most consistently separating COO-unclassified DLBCL from either ABC- or GCB-DLBCL are illustrated below with cluster membership and mean differential expression rank shown. See corresponding Additional file 11

We next addressed to what extent the identified polarized pattern of immune response was selective for COO-unclassified DLBCL or whether equivalently intense expression of polarized immune response genes might be detectable amongst some DLBCL cases that could be assigned to a principal COO class. As noted above, the COO-unclassified meta-profile separated on hierarchical clustering from the gene perspective into two branches, one of which was more strongly linked to core T-cell and cytotoxic genes (Fig. 4). To examine the relative ranking of genes belonging to these two hierarchical clustering branches within the COO-unclassified meta-profile we superimposed the cluster membership onto scatter plots of differential expression ranking. We first ranked and then plotted genes belonging to the meta-profile by median fold differential expression in the comparison of COO-unclassified with ABC- or GCB-DLBCL. This demonstrated a significant overall correlation in the differential expression of COO-unclassified meta-profile genes relative to either principal COO class. Furthermore, genes belonging to the “T-cell cluster” (cluster 1) were significantly skewed toward most consistent association with COO-unclassified DLBCL (Additional file 11). To address whether the consistency of differential detection between data sets would alter this conclusion we ranked genes by a measure derived from both the number of data sets (consistency of differential expression) in which a gene was differentially expressed and the normalised median fold differential expression (Additional file 12). This again showed a significant overall correlation and a skewing of the T-cell cluster toward most consistent association with COO-unclassified DLBCL (*p* = 6.57E-06, hypergeometric test; Fig. 4). However, using either approach *IFNG* was identified as amongst the cluster 2 genes most consistently linked to COO-unclassified DLBCL.

**Fig. 5:**
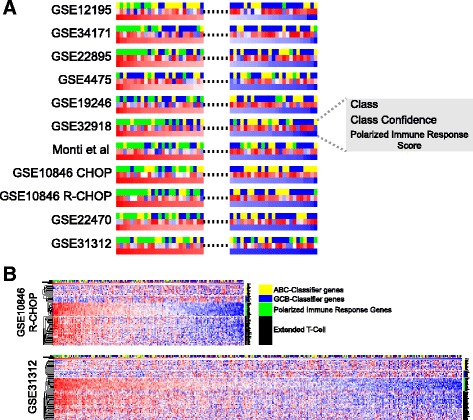
The polarized immune response is a dominant feature across DLBCL, independent of COO class. **a** The incidences of individual cases across all data sets (note GSE10846 is subdivided into CHOP and R-CHOP treated components) ranked according to polarized immune response score. The top and bottom 25 cases for each data set are illustrated with colour coding for COO class shown in the *top bar* (*yellow* ABC, *blue* GCB, *green* unclassified), class confidence assigned during classification shown in the *middle bar* (*blue* low confidence to *red* high confidence), and polarized immune response score shown in the *bottom bar* (*blue* low polarized immune response score to *red* high polarized immune response score). **b** Complete results for data sets GSE10846 R-CHOP and GSE31312, showing all cases ranked by polarized immune response score. Each heatmap displays class assignment, classification confidence and polarized immune response score summary as in (**a**) followed by COO-classifier gene expression (*yellow* and *blue bars*), the 16 genes of the polarized immune response score (*green bar*), and the extended set of COO-unclassified meta-profile genes (*black bar*). A corresponding high-resolution figure comprising equivalent representation for all data sets is provided in Additional file 13

To examine the contribution of polarized immune response genes associated with COO-unclassified DLBCL across all data sets on a case-by-case basis we developed a linear additive classifier. For this we employed the 16 genes most strongly linked to COO-unclassified DLBCL derived from analysis using both the consistency/data set number and median fold differential expression. Given the contribution of core T-cell elements, cytotoxic genes and *IFNG*, we consider this to represent an integrated assessment of a polarized immune response. We ranked all cases in each data set by this linear score and plotted the incidence of cases classified as ABC, GCB and unclassified on this ranking. Overall, individual COO-unclassified DLBCL cases showed a stronger association with the polarized immune response score relative to either ABC- or GCB-DLBCL (Fig. 5a; Additional file 13). This was particularly evident in the larger data sets GSE31312, GSE22470 and GSE10846. However, ABC- and GCB-DLBCL cases with high levels of expression of the polarized immune response score were present in all data sets.

To assess whether the 16-gene score also reflected the expression of other genes associated with the immune response in COO-unclassified DLBCL we added further components of the meta-profile. Expression of these genes followed the overall pattern of expression of the 16-gene score across all DLBCL data sets (Fig. 5b; Additional file 13). Thus, the 16-gene score provides a tool with which to identify the overall pattern of this polarized immune response in DLBCL.

**Fig. 6:**
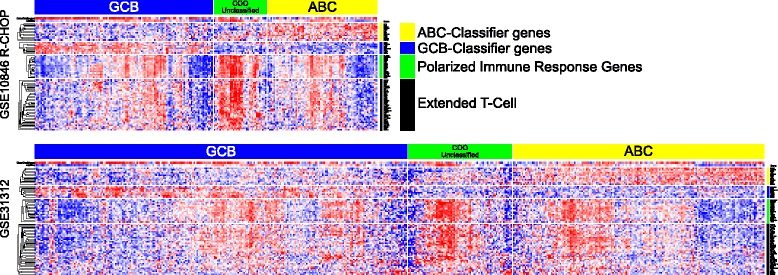
The polarized immune response subdivides COO-unclassified DLBCL and identifies subsets of cases within ABC- and GCB-DLBCL classes. Heatmaps illustrate data for GSE10846 R-CHOP and GSE31312 hierarchically clustered according to all genes shown, and constrained by COO class assignment. Assigned COO class is shown above each heat map by the *blue* (GCB), *green* (COO-unclassified) and *yellow* (ABC) bars. To the right is shown the corresponding general category of genes: *yellow* ABC-classifier genes, *blue* GCB-classifier genes, *green* polarized immune response score genes, and *black* extended COO-unclassified meta-profile. A corresponding high-resolution figure comprising equivalent representation for all data sets is provided in Additional file 14

Since some COO-unclassified DLBCL cases in all data sets showed low polarized immune response scores, we examined the pattern of T-cell gene expression further by hierarchical clustering within each COO class. This demonstrated, particularly in the larger data sets such as GSE31312 and GSE22470, that COO-unclassified DLBCL could be segregated into principal groups with a subset of cases characterized both by weak expression of COO-classifier genes and weak expression of polarized immune response genes (Fig. 6; Additional file 14). Within the ABC- and GCB-DLBCL subsets there was a common concordance between expression of core T-cell genes and components of the polarized immune response. Only a few cases, particularly in the GCB-DLBCL subset, could be identified in which core T-cell genes were co-expressed in the absence of other elements of the polarized response. These cases were, however, too few to allow meaningful analysis (data not shown). Thus, across all DLBCL data sets the expression of core T-cell genes is paralleled by the expression of genes linked to functional polarization irrespective of COO class.

### Polarised immune response and COO-unclassified DLBCL do not overlap significantly with signatures of primary mediastinal B-cell lymphoma

COO-unclassified DLBCL cases lacking both polarized immune response and COO-classifier gene expression are distinct from the subset of cases in which the extent of the polarized immune response obscures the characterization of the neoplastic B-cell population. At least two principal explanations could be considered for this subgroup: on the one hand these might include cases in which gene expression was technically challenging with poor representation of tumour cell RNA; alternatively, they might include a subset of large B-cell lymphoma which fails to express COO-classifier genes at significant levels. Primary mediastinal B-cell lymphoma (PMBL) is a biologically distinct subgroup of large B-cell lymphoma, more common in women, with a mediastinal localization, distinct molecular genetics and possible derivation from a thymic B-cell population [47]. This lymphoma class can be associated with a pattern of gene expression distinct from either GCB- or ABC-DLBCL. While many PMBL cases would be excluded on the basis of diagnosis from conventional DLBCL gene expression data sets, it was possible that some PMBL cases might contribute to the COO-unclassified DLBCL cases, in particular those lacking a polarized immune response signature. To address this we used the 23-gene PMBL signature described by Rosenwald et al. [40], and first tested for enrichment within the COO-classified and COO-unclassified meta-profiles, but this showed no evidence of significant enrichment, nor was a signature separating PMBL from Hodgkin lymphoma enriched (Additional file 6). We next used the 23-gene PMBL signature in place of the extended immune response gene list to reanalyse the DLBCL data sets by hierarchical clustering (Additional file 15). We found no evidence of distinct clusters of cases identifiable with the 23-gene PMBL signature amongst COO-unclassified DLBCL, although a few elements of the 23-gene signature, most notably *PDCD1LG2*, *CD274* and *BATF3*, do correlate with the polarized immune response. In contrast, in several data sets small clusters of cases were identifiable with coordinated high expression of the 23 genes of the PMBL signature, but such cases were classifiable as GCB-DLBCL, suggesting a greater overlap of PMBL signature gene expression amongst cases otherwise classifiable as GCB-DLBCL rather than ABC-DLCBL or COO-unclassified DLBCL. Thus, we found no gene expression-based evidence for a significant contribution of PMBL-like gene expression patterns amongst COO-unclassified DLBCL in the data sets analysed. Inclusion of PMBL-like cases does not have a major impact on the detection of the polarized immune response signature, nor provide an explanation for the subset of COO-unclassified DLBCL that lacks both COO-classifier and polarized immune response gene expression.

### A polarized immune response is associated with improved outcome in DLBCL

**Fig. 7:**
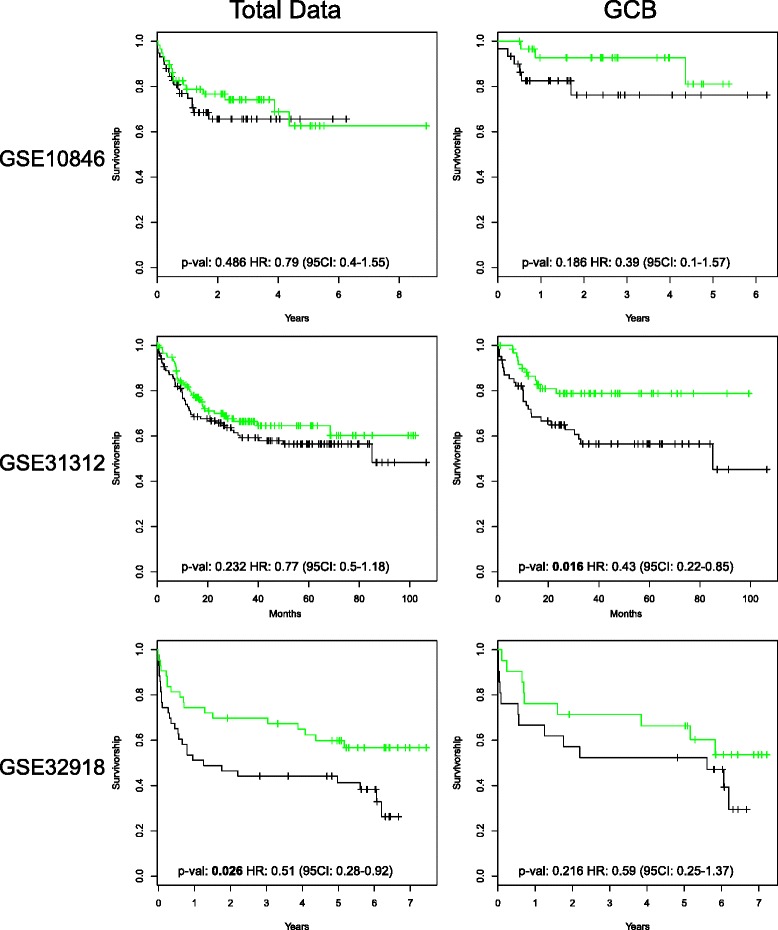
A high polarized immune response score is associated with improved outcome in R-CHOP-treated GCB-DLBCL. The figure illustrates Kaplan–Meier plots of overall survival derived from R-CHOP-treated DLBCL cases from data sets GSE10846, GSE31312 and GSE32918. Illustrated is the overall survival for the top and bottom 25 % of cases divided by polarized immune response score. The *left graphs* illustrate results independent of COO class and the *right graphs* results for cases assigned to the GCB-DLBCL class. *CI* confidence interval, *HR* hazards ratio

Across several cancer types the extent of tumour infiltrating lymphocytes, and their polarization toward cytotoxic T/natural killer (NK) cell gene expression linked to an IFNγ-STAT1-IRF1 signalling axis has been identified as a feature associated with good prognosis [4]. We therefore asked whether the expression of the polarized immune response signature, alone or taken in conjunction with COO class, was associated with differences in overall survival. Currently DLBCL is treated with an immunochemotherapy regimen, R-CHOP, which combines the anti-CD20 therapeutic monoclonal antibody rituximab with cyclophosphamide, hydroxydaunorubicin, vincristine (Oncovin), and prednisolone. Based on the success of the R-CHOP regimen, current treatment and future therapeutic trials in DLBCL will be based on immunochemotherapeutic approaches encompassing rituximab or related therapeutic antibodies. Therefore, only those data sets (GSE10846, GSE31312 and GSE32918) encompassing R-CHOP-treated cases associated with appropriate survival data were considered. This analysis demonstrated a consistent trend toward a reduced hazard ratio of death with increasing polarized immune response score across all three R-CHOP-treated DLBCL data sets. This reached statistical significance when considered independently of COO class in data sets GSE32918 and GSE31312, the latter representing the largest data set of R-CHOP-treated DLBCL [23]. However, in these two data sets the polarized immune response score was also significantly associated with lower age. When considered according to COO classification a consistent trend toward better outcome with high polarized immune response score was observed across all three categories. This trend was most pronounced for GCB-DLBCL, and reached statistical significance for improved outcome associated with high polarized immune response score in the largest data set GSE31312 (Additional file 16; Fig. 7). We conclude, therefore, that the presence of a polarized and IFNγ-associated immune response shows an association with good outcome which is modified by consideration of COO class, such that in the context of current R-CHOP therapy a polarized immune response is most consistently linked to improved outcome in patients with GCB-DLBCL.

### Polarization along an IFNγ-STAT1-IRF1 axis is a defining feature of the DLBCL immune response

**Fig. 8:**
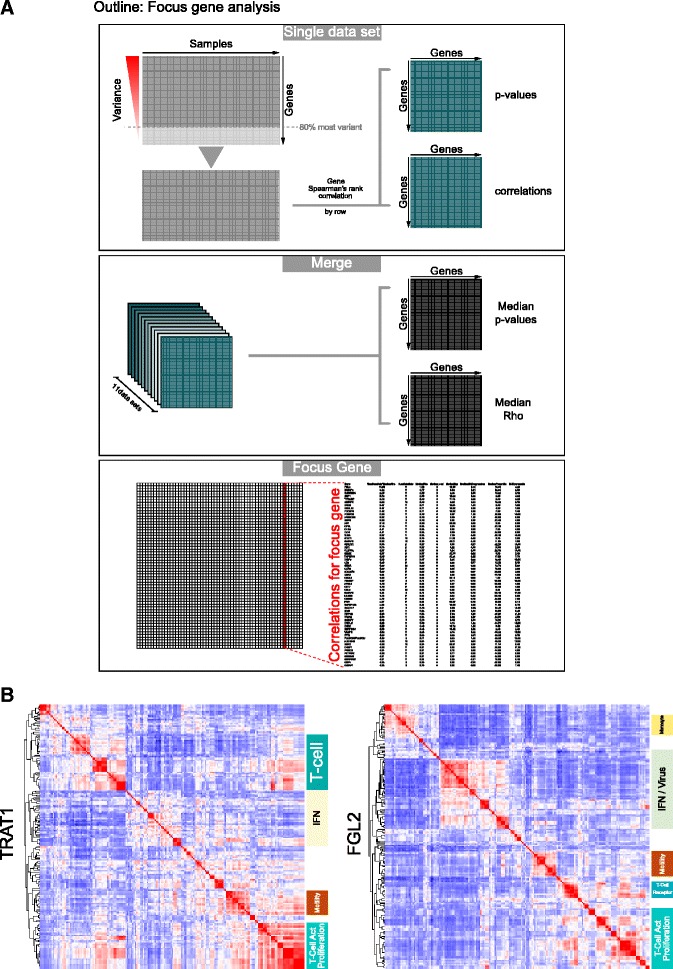
A focus gene analysis independent of COO class verifies the dominant polarized immune response in DLBCL. **a** An outline of the focus gene analysis (high resolution version in Additional file 3). *Upper panel*: the approach within each data set, with initial selection of the 80 % most variable genes, and subsequent generation of linked matrices of gene correlation values and associated *p* values. *Middle panel*: the merging of all data sets (11 data sets; data set GSE10846 subdivided by treatment type) is shown to give matrices of median correlations and *p* values. *Lower panel*: the selection of an individual focus gene for downstream analysis. **b** Results of integrated gene signature and ontology analysis for two focus genes (*left panel TRAT1*) and (*right panel FGL2*) displaying the clustering of enriched signature and GO terms. General terms corresponding to major correlation clusters are illustrated to the right of each heatmap. Corresponding high resolution versions are available in Additional files 18 and 19, which also include the corresponding heatmaps clustered from the gene perspective

While the above analysis pointed to a common convergence onto a cytotoxic and IFNγ-polarized immune response in DLBCL, not all components of the IFNγ-STAT1-IRF1 axis were sufficiently differentially expressed between COO-classified and COO-unclassified DLBCL to be identified by this approach. In order to explore the DLBCL-associated immune response in a fashion which was not constrained by the COO paradigm we re-analyzed the DLBCL data sets, assessing the consistency and degree of correlated gene expression across all data sets relative to a selected “focus gene” (Fig. 8a; Additional files 3 and 4). We followed this by applying the integrated signature and GO enrichment analysis (Additional file 17).

As focus genes we selected two components of the 16-gene polarized immune response signature, *TRAT1* and *FGL2*, to reflect origin from the two branches of the COO-unclassified meta-profile (Fig. 8b; Additional files 18 and 19). *TRAT1* was selected as the most highly correlated gene from cluster 1 (Fig. 4), while *FGL2* was selected as the second most highly correlated gene in cluster 2, and of more established immunologic interest than *TC2N* and less overt connection to immune response polarization than *IFNG*, the other two genes derived from cluster 2 that contribute to the 16-gene polarized immune response classifier.

Genes correlating with *TRAT1* could be assigned to clusters of signatures and GO terms related to T-cell state, and T-cell signal transduction, cell motility and interferon response. For *FGL2* as the focus gene a similar pattern emerged, including an expanded cluster of signature enrichments related to interferon responses, including some derived from models of viral infection, and an additional association with monocyte/macrophage-derived signatures.

**Fig. 9:**
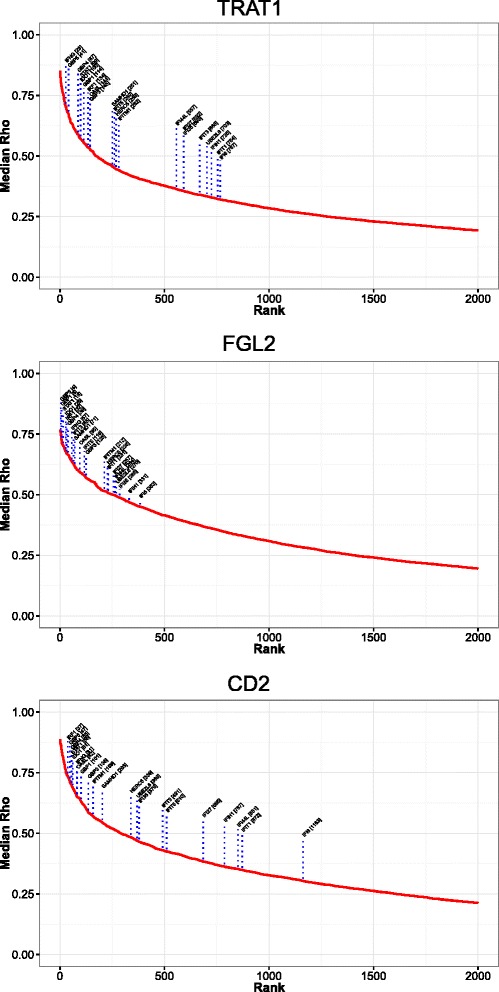
IFN-responsive genes and the IFNγ-STAT1-IRF1 axis are amongst the leading edge of highly correlated DLBCL immune response genes. Correlation curves were generated from the focus gene analysis by ranking genes according to median correlation, and then plotting the gene correlation rank (x-axis) against the corresponding median gene correlation (y-axis, median Rho). This illustrates both the relative strength of correlations for each focus gene and identifies a leading edge of genes with most significant correlations. The position of a set of IFN-associated genes was plotted for each focus gene context as indicated in the figure. Note only the top 2000 of 20,121 genes tested are illustrated. See corresponding Additional file 20

To examine the strength of correlation with IFN-responsive genes we ranked genes by median correlation, plotted rank against median gene correlation for each focus gene context and assessed the distribution of selected IFN signature genes (derived from the previous analysis) on the resulting correlation curves. We applied this approach using *TRAT1* and *FGL2* as focus genes, but observed similar results with all 16 genes of the polarised immune response classifier (Fig. 9; Additional files 20). In either context IFN pathway genes were consistently present within the leading edge of most correlated genes, including *IFNG*, *STAT1*, *IRF1*, *GBP1*, *GBP5* and *IDO1*. These genes were also consistently present within the leading edge when considering a more generic T/NK cell-associated gene, *CD2*, as focus gene. Components of the IFNγ-STAT1-IRF1 axis therefore emerge as a consistent and dominant feature of the DLBCL immune environment linked to expression of a wider complement of IFN-responsive genes.

### IFNγ-STAT1-IRF1 axis and adaptive immune regulatory pathways in DLBCL

**Fig. 10:**
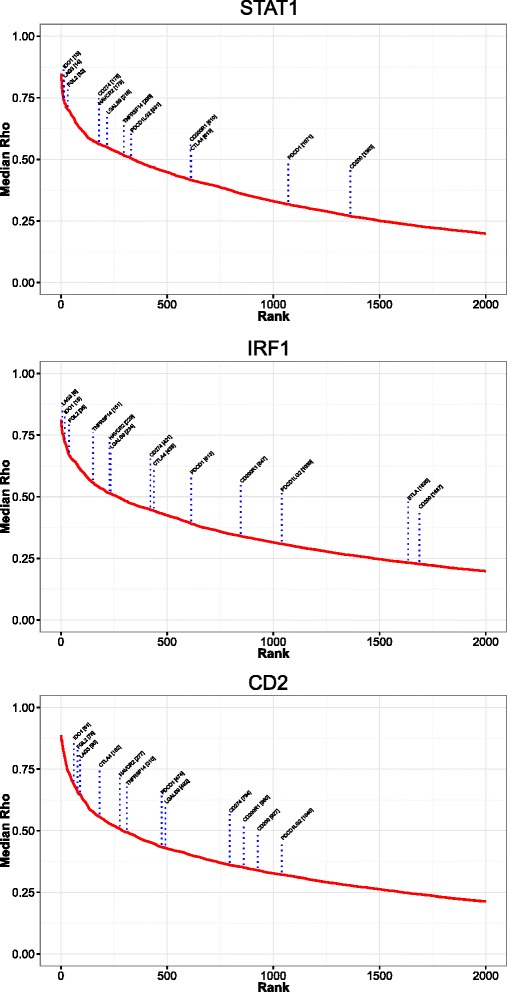
Immune-modulatory and checkpoint gene expression is strongly correlated with elements of the IFNγ-STAT1-IRF1 axis in DLBCL. *IRF1* and *STAT1* along with *CD2* were analysed as focus genes, and resultant correlation curves are illustrated. Genes were plotted according to correlation rank (x-axis) against median gene correlation (y-axis, median Rho). The position of immune checkpoint/modulatory genes on the resulting curves was plotted for each focus gene as indicated in the figure. Note only the top 2000 of 20,121 genes tested are illustrated. See corresponding Additional file 21

In the model of adaptive immune resistance IFNγ-driven expression of PD1 ligands *CD274* and *PDCD1LG2* on tumour cells and the microenvironment limits local T-cell responses [5, 8]. We reasoned that the hierarchy of gene expression correlations would allow a ranking of immune checkpoint/modulatory gene expression linked to the IFNγ-STAT1-IRF1 polarized response in DLBCL. In this pathway *STAT1* and *IRF1* encode the transcriptional regulators; we therefore selected these along with *CD2* as a generic representative of the T/NK cell response for analysis (Fig. 10; Additional file 4). When considering immune modulatory/checkpoint genes a consistent cluster of three genes, *LAG3*, *IDO1* and *FGL2*, emerged as most highly ranked and amongst the leading edge in all three focus gene contexts. In contrast, *CD274* and *PDCD1LG2* showed significantly weaker correlations with each focus gene, but nonetheless remained well correlated in comparison with all genes tested (rank <1000 out of 20,121 tested). To further confirm this pattern we extended the analysis to all 16 genes of the polarized immune response classifier, and observed similar patterns of gene correlation ranking (Additional file 21). Since the relative contribution of immune modulatory/checkpoint gene expression in tumour cells themselves relative to the wider microenvironment cannot be determined from these analyses, we conclude that, in addition to *CD274* and *PDCD1LG2*, a wider complement of immune modulators provides a potentially high degree of redundancy in adaptive immune resistance in DLBCL. Amongst these components *IDO1*, *FGL2* and *LAG3* are particularly strongly correlated with IFNγ polarized immune responses.

## Discussion

The common convergence of cancer immune responses onto patterns of cytotoxic and IFNγ-dominated pathways has been summarised in the concept of an “immune constant of rejection” [4, 7]. In parallel the model of adaptive immune resistance argues for the control of such immune responses via local feedback driven through IFN-mediated upregulation of immune checkpoints [5, 8]. Our analysis here provides extensive empirical evidence across currently available large DLBCL data sets that this combination of IFNγ polarisation and induction of adaptive immune resistance mechanisms is a feature of the immune response to DLBCL. Unbiased analysis of gene expression correlations moreover suggests a hierarchy of IFN-associated immune modulatory gene expression with *LAG3*, *IDO1* and *FGL2* as key elements in this context. Thus, adaptive immune resistance is likely to provide an important component of immune evasion in DLBCL.

Other mechanisms of immune evasion have been previously identified as playing an important role in the pathogenesis of DLBCL, including mutation and deletion of *B2M* and *CD58*, and amplification of genomic regions encompassing genes encoding PD1 ligands [48, 49]. Furthermore previous studies have demonstrated the presence of PD1 expression on infiltrating T-cell populations and PD-L1(CD274) on tumour cells and in the microenvironment of DLBCL and related neoplasms [50, 51]. In the context of gene expression profiling, morphologically defined T-cell and histiocyte-rich large B-cell lymphoma, which represents a relatively rare subcategory, has been characterized by evidence of an IFN-associated immune response, linked on the one hand with over-expression of *PD1* (*PDCD1*) on infiltrating T cells when compared with classical Hodgkin lymphoma [52], or the expression of *IDO1* when compared with nodular lymphocyte predominant Hodgkin lymphoma, another relatively rare lymphoma subtype [53]. Indeed, expression of IDO1 has been defined as a feature associated with poor outcome in generic DLBCL in one patient series [54]. Thus, the involvement of several pathways of immune modulation in large B-cell lymphomas is supported by prior studies.

Using the 16-gene polarized immune response score we have ranked DLBCL cases across multiple data sets, and demonstrate that a substantial fraction of cases regardless of COO class are linked to a polarized immune response. In the context of the COO classification, the dominance of this immune response at the expense of proliferating B cells provides the most common explanation for DLBCL cases that are “unclassifiable” as originally suggested by Monti et al. [15]. Equally important is the identification of a distinct group of DLBCL characterized by an absence of host response elements, which is consistent with “immunological ignorance”, a feature which in other cancers is associated with poor response to immune checkpoint blockade [12]. These cases are also consistent with a model of host tissue “effacement” proposed by Scott and Gascoyne [49] as distinguishing subsets of aggressive lymphomas. Immune evasion in DLBCLs can be associated with loss of MHC class I expression consequent on mutation and/or deletion of *B2M*, which may be further accompanied by inactivation of *CD58* [48], and a prediction might be that such cases would be enriched in the subset characterized by apparent immunological ignorance. However, analogous lesions affecting *B2M* were recently identified as recurrent events positively associated with cytotoxic gene signatures in solid tumours [9]. This suggests a model in which adaptive immune resistance mechanisms may be followed by somatic genetic alterations that further enhance tumour immune escape. Whether a similar positive association between cytotoxic response and *B2M* or *CD58* mutation status exists in DLBCL is, to our knowledge, not established.

Across several cancer types the intensity of tumour infiltrating lymphocytes and their functional polarization has proved to be of prognostic significance in the absence of specific immune checkpoint blockade [4, 55–57]. Our analysis indicates that a trend toward an improved outcome in association with a more intense polarized immune response is generally maintained in the context of DLBCL treated with the current immunochemotherapy regimen, R-CHOP. However, this benefit is not equivalent across all DLBCL when considered in relation to COO class, and is most pronounced for GCB-DLBCL. Indeed, in the largest available data set of R-CHOP-treated DLBCL, GSE31312 [23], a substantial group of patients with both a GCB-DLBCL classification and a high polarized immune response score appeared curable with current therapy. As a statistically significant association is not consistently observed across all three data sets of DLBCL treated with R-CHOP, and there is a potentially confounding association with young age, the overall prognostic value of this association remains uncertain in the context of current therapy. Additional features of the host response, which did not emerge as principal discriminants between COO-classified versus COO-unclassified DLBCL, such as contributions from macrophage/monocyte lineage cells, may add value to immune response classifiers. These will need to be considered alongside the polarized immune response signature in future work. Nonetheless, the analysis presented here demonstrates a graded pattern of immune response in DLBCL, with one extreme characterized by minimal cytotoxic immune response signature and tendency to poor outcome, and another extreme characterized by intense polarized immune response and a tendency toward better outcome which is modified by COO class. In other settings the pattern of pre-existing immune response prior to immune checkpoint therapy has proved to be of predictive value [11, 12, 58, 59]. Based on this evidence, it is the subset of DLBCL cases with pre-existing polarized immune response which is most likely to benefit from immune checkpoint/modulatory therapy, and is readily identifiable in a quantitative fashion from gene expression data.

Immune checkpoint inhibitors are already under evaluation in the context of large cell lymphomas [60, 61]. Recent clinical trials with PD1 pathway blockade have shown substantial promise in Hodgkin lymphoma [14], as in other tumour types [11, 12, 62]. Combining immune checkpoint inhibitors may hold particular promise, and both LAG3 and IDO1 are therapeutic targets with novel agents in current clinical evaluation. Our analyses support these as high priority candidates for therapeutic evaluation in DLBCL alongside PD1 blockade. In addition to direct interventions specifically targeting immune checkpoints, signalling pathways that mediate survival of neoplastic B cells, and are the targets of novel therapeutic agents in lymphoma, overlap with pathways controlling T-cell responses. Such agents have the potential to de-repress cytotoxic T-cell populations and promote anti-tumour immunity [63]. Thus, companion biomarkers evaluating the potential association between pre-existing immune response at diagnosis and treatment response should arguably also be included in the setting of lymphoma clinical trials where agents targeting lymphocyte signalling pathways are being evaluated.

A notable element of the DLBCL immune response is the consistent association with *FGL2* expression. This encodes fibrinogen-like 2 prothrombinase, a protein that has dual roles as a pro-coagulant and immune modulator. FGL2 has been shown to act as an immune responsive coagulant in settings such as foetal loss driven by Th1 polarized immune responses [64] and fulminant hepatitis [65]. Subsequently, FGL2 has been implicated as a repressor of T-cell activation both in the ability of recombinant FGL2 to block graft rejection [66] and in the context of *Fgl2* knockout mice developing autoimmune glomerulonephritis [67]. In several experimental models FGL2 has been associated with suppression of cytotoxic and Th1-polarized immune responses [67–69]. FGL2 effects in DLBCL could relate to both pro-coagulant and immune modulatory functions. In DLBCL *FGL2* expression correlates with multiple elements of the IFNγ-STAT1-IRF1 axis; supporting direct regulation, FGL2 expression has previously been shown to be responsive to IFNγ in T cells [70, 71], and was shown to act downstream of IRF1 in Th1-driven foetal loss [64]. Thus, the relationships in DLBCL suggest that FGL2 may provide an additional element of negative feedback and adaptive immune resistance, which is potentially suitable for therapeutic targeting [72, 73].

We note that some DLBCL cases with a prominent immune response may be associated with Epstein-Barr virus (EBV) infection and oncogenic drive. In the meta-analysis approach taken here the contribution of EBV cannot be systematically assessed from available data since EBV status is incompletely annotated, and not necessarily assessed using both immunohistochemistry for EBV LMP1 and RNA-FISH for EBERs. Immune surveillance is known to contribute to the control of EBV-mediated tumours [74], and the presence of high EBV loads can contribute to the establishment of an exhausted cytotoxic response [75]. Indeed, there are significant overlaps between the gene expression profiles of the immune response in EBV-associated large cell lymphomas occurring in the post-transplant setting [76] and the polarized IFNγ-associated gene expression that is evident from our DLBCL meta-analysis. However, while the frequency of EBV infection in generically diagnosed DLBCL has been established at close to 10 % [77], significant expression of genes linked to the polarized immune response is more frequent across DLBCL data sets. An overlap of gene expression profiles between the immune response targeting EBV-driven and EBV-independent lymphomas is consistent with the model of convergent patterns of “immune rejection” across diverse immune contexts [4, 7]. It is arguable that the principal predictive factor of response to immune checkpoint inhibition will be the presence of a pre-existing polarized immune response and the mechanisms controlling its chronic activation/exhaustion rather than the nature of the initial triggering antigens whether viral or cancer-associated.

## Conclusions

The analysis presented here supports the central importance of convergent patterns of immune response linked to the IFNγ-STAT1-IRF1 axis, coupled to IFN-driven feedback pathways in DLBCL. This argues for the generalisable nature of these interconnected mechanisms, and implicates a hierarchy of immune modulators, known to promote the establishment of an immunosuppressive microenvironment [2], in the process of IFNγ-driven adaptive immune resistance.

## Additional files

Additional file 1: Figure S1. Outline of meta-profile generation using COO classification. *Upper panel*: the data sets used. Please note one data set, GSE10846, is divided into two component parts reflecting underlying differences in treatment (CHOP versus R-CHOP), giving a total of 11 separate data set components. Illustrated are all cases for each data set, subdivided by COO classification established using the DAC classifier [32] and ranked by classification confidence, with classifier genes illustrated on the right (*yellow bars* ABC classifier genes and cases, *blue bars* GCB classifier genes and cases, *green bars* COO-unclassified cases). *Middle panels*: the pairwise comparisons of differentially expressed genes between three classes, and the integration of differentially expressed genes across data sets. *Bottom panels*: the resulting meta-profiles of differentially expressed genes for both components of the three possible pairwise comparisons are shown as Wordles for illustrative purposes (complete details provided in Additional file 5). (PDF 2024 kb) Additional file 2: Figure S2. Outline of the process for integrating and visualizing analysis of gene signature and ontology enrichments. The flow diagram illustrates the process for integrating gene signature and ontology enrichments. The initial assessment of overlap between meta-profiles derived from the comparison of ABC-DLBCL versus COO-unclassified (*CU*) DLBCL and GCB-DLBCL versus COO-unclassified (*CU*) DLBCL is shown at the top of the figure, followed by the parallel analysis of gene ontology (BiNGO) and hypergeometric testing of signature enrichments. Next a matrix is illustrated showing the occurrence of genes versus enriched signatures (*green fill*), followed by analysis of correlations (Phi coefficient) by column (signature/ontology terms) or by row (genes) and hierarchical clustering. (PDF 1658 kb) Additional file 3: Figure S11. An outline of the focus gene approach, and a high resolution image to accompany Fig. 8a. *Upper panel*: the approach within each data set with initial selection of the 80 % most variable genes, and subsequent generation of linked matrices of gene correlation values and associated *p* values. *Middle panel*: merging of all data sets (11 data sets; data set GSE10846 subdivided by treatment type) to give gene by gene matrices of median correlations and *p* values. *Lower panel*: the selection of an individual focus gene for downstream analysis. (PDF 1375 kb) Additional file 4: Table S5. Lists of correlated genes for selected focus genes. (XLSX 16708 kb) Additional file 5: Table S1. Lists of Meta-profile genes differentially expressed between DLBCL COO classes. (XLSX 2318 kb) Additional file 6: Table S2. Lists of enriched gene signature and gene ontology terms for COO-classified and COO-unclassified meta-profiles. (XLSX 2848 kb) Additional file 7: Figure S3. High resolution image corresponding to Fig. 2a. Integrated gene signature and ontology enrichments for COO-classified meta-profile clusters from signature and ontology term perspectives. The figure represents the hierarchical clustering of enriched gene signature and ontology terms related to the COO-classified meta-profile. Correlations are illustrated in heat maps on a *blue* (least) to *red* (most) scale as indicated at the top of the figure. Along the edges of the heatmap the signature terms are provided (and correspond to terms listed in Additional file 6). The FDR-corrected *p* value for enrichment of the signature is illustrated as a bar on either side of the heatmap, along with an indication of the type of term (signature versus ontology) and the origin of the terms as indicated in the figure. (PDF 283 kb) Additional file 8: Figure S4. High resolution image corresponding to Fig. 2b. Integrated gene signature and ontology enrichments for COO-classified meta-profile clustered from the gene perspective. The figure represents the hierarchical clustering of meta-profile genes contributing to signature and ontology term enrichments, and clustered according to the correlation of enriched signature/ontology term membership. Correlations are illustrated in the heatmap on a *blue* (least) to *red* (most) scale as indicated at the top of the figure. Along the edges of the heatmap official gene symbols are provided. (PDF 251 kb) Additional file 9: Figure S5. High resolution image corresponding to Fig. 3a. Integrated gene signature and ontology enrichments for COO-unclassified meta-profile clusters from signature and ontology term perspectives. The figure represents the hierarchical clustering of enriched gene signature and ontology terms related to the COO-unclassified meta-profile. Correlations are illustrated in heat maps on a *blue* (least) to *red* (most) scale as indicated at the top of the figure. Along the edges of the heatmap the signature terms are provided (and correspond to terms listed in Additional file 6). The FDR-corrected *p* value for enrichment of the signature is illustrated as a bar on either side of the heatmap, along with an indication of the type of term (signature versus ontology) and the origin of the terms as indicated in the figure. (PDF 518 kb) Additional file 10: Figure S6. High resolution image corresponding to Fig. 3b. Integrated gene signature and ontology enrichments for COO-unclassified meta-profile clustered from the gene perspective. The figure represents the hierarchical clustering of meta-profile genes contributing to signature and ontology term enrichments, and clustered according to the correlation of enriched signature/ontology term membership. Correlations are illustrated in the heatmap on a *blue* (least) to *red* (most) scale as indicated at the top of the figure. Along the edges of the heatmap official gene symbols are provided. (PDF 126 kb) Additional file 11: Figure S7. Relates to Fig. 4. Genes most consistently associated with COO-unclassified DLBCL are related to a polarized immune response. As in Fig. 4, the two principal branches of the gene-centred hierarchical clustering tree of the COO-unclassified meta-profile are illustrated on the *left*. Colour-coding above identifies: *red* cluster 1, corresponding to the T-cell cluster; *black* cluster 2, IFN and monocyte/immune *NOS* (not otherwise specified). On the right the relative rank of differentially expressed genes contributing to the COO-unclassified meta-profile is plotted using the median normalized fold change for gene ranking; the x-axis indicates differential expression rank in the comparison COO-unclassified versus ABC-DLBCL; the y-axis indicates differential expression rank in the comparison COO-unclassified versus GCB-DLBCL. Cluster membership is superimposed on the scatter plot of differential expression rank according to the colour coding shown (*red* cluster 1, *black* cluster 2). The 18 genes most consistently separating COO-unclassified DLBCL from either ABC- or GCB-DLBCL are illustrated below with cluster membership and mean differential expression rank shown. (PDF 275 kb) Additional file 12: Table S3. Ranked list of genes contributing to shared meta-profiles of COO-classified and COO-unclassified DLBCL. (XLSX 38 kb) Additional file 13: Figure S8. Relates to Fig. 5. Ranking by the 16-gene polarized immune response score demonstrates common occurrence of a polarized T-cell response across DLBCL from all data sets. Shown are all DLBCL data sets used with cases ranked by the 16-gene polarized immune response score. The data set number is shown above each heat map, followed by three bars: *top bar* COO class (*yellow* ABC, *blue* GCB, *green* unclassified); *middle bar* class confidence assigned during classification (*blue* low confidence to *red* high confidence); *bottom bar* polarized score (*blue* low polarized immune response score to *red* high polarized immune response score). These are followed by case-by-case gene expression values (illustrated as z scores) which are broken down into components identified by coloured bars on the right of each heatmap. The contributing genes are shown in the *grey expanded box* to the right of the figure with corresponding colour code: *yellow bar* ABC COO-classifier genes; *blue bar* GCB COO-classifier genes; *green bar* polarized immune response score; *black bar* extended COO-unclassified meta-profile and immune response genes. (PDF 1073 kb) Additional file 14: Figure S9. Relates to Fig. 6. Clustering within COO classes demonstrates subdivision of unclassified DLBCL by polarized immune response score, and occurrence of immune response-rich cases in each principal COO class. Shown are all DLBCL data sets used, hierarchically clustered by all genes shown, and constrained by COO class. The data set number is shown above each heatmap, followed by three bars: *top bar* COO class (*yellow* ABC, *blue* GCB, *green* unclassified); *middle bar* class confidence assigned during classification (*blue* low confidence to *red* high confidence); *bottom bar* polarized score (*blue* low polarized immune response score to *red* high polarized immune response score). These are followed by case-by-case gene expression values (illustrated as z scores), which are broken down into components identified by coloured bars on the right of each heatmap. The contributing genes are shown in the *grey expanded box* to the right of the figure with corresponding color code: *yellow bar* ABC COO-classifier genes; *blue bar* GCB COO-classifier genes; *green bar* polarized immune response score; *black bar* extended COO-unclassified meta-profile and immune response genes. (PDF 1168 kb) Additional file 15: Figure S10. Clustering of COO classes and PMBL signature genes. Shown are all DLBCL data sets used, hierarchically clustered by all genes shown, including the 23-gene PMBL signature, and constrained by COO class. The data set number is shown above each heatmap, followed by three bars: *top bar* COO class (*yellow* ABC, *blue* GCB, *green* unclassified); *middle bar* class confidence assigned during classification (*blue* low confidence to *red* high confidence); *bottom bar* polarized score (*blue* low polarized immune response score to *red* high polarized immune response score). These are followed by case-by-case gene expression values (illustrated as z scores), which are broken down into components identified by coloured bars on the right of each heatmap. The contributing genes are shown in the *grey expanded box* to the right of the figure with corresponding color code: *yellow bar* ABC COO-classifier genes; *blue bar* GCB COO-classifier genes; *green bar* polarized immune response score; *black bar* PMBL 23-gene signature. (PDF 1002 kb) Additional file 16: Table S4. Details for survival/outcome data related to the linear score. (XLSX 17 kb) Additional file 17: Table S6. Signature and ontology enrichments for the leading edge of most correlated genes for each focus gene context. (XLSX 2731 kb) Additional file 18: Figure S12. Relates to Fig. 8b. Integrated gene signature and ontology enrichments for *TRAT1* focus gene analysis. *Upper panel*: the hierarchical clustering of enriched gene signature and GO terms related to genes correlating with *TRAT1* in focus gene analysis. Correlations are illustrated in the heatmap on a *blue* (least) to *red* (most) scale as indicated at the top of the figure. Along the edges of the heatmap the signature terms are provided (and correspond to terms listed in Additional file 17). The FDR-corrected *p* value for enrichment of the signature is illustrated as a bar on either side of the heatmap, along with an indication of the type of term (signature versus ontology) and the origin of the terms as indicated in the figure. *Lower panel*: the hierarchical clustering of genes correlating with *TRAT1* in focus gene analysis. Genes shown are clustered according to the correlation of enriched signature/GO term membership. Correlations are illustrated in the heatmap on a *blue* (least) to *red* (most) scale as indicated at the top of the figure. Along the edges of the heatmap official gene symbols are provided. (PDF 1104 kb) Additional file 19: Figure S13. Relates to Fig. 8b. Integrated gene signature and ontology enrichments for *FGL2* focus gene analysis. *Upper panel*: the hierarchical clustering of enriched gene signature and GO terms related to genes correlating with *FGL2* in focus gene analysis. Correlations are illustrated in the heatmap on a *blue* (least) to *red* (most) scale as indicated at the top of the figure. Along the edges of the heatmap the signature terms are provided (and correspond to terms listed in Additional file 17). The FDR-corrected *p* value for enrichment of the signature is illustrated as a bar on either side of the heatmap, along with an indication of the type of term (signature versus ontology) and the origin of the terms as indicated in the figure. *Lower panel*: the hierarchical clustering of genes correlating with *FGL2* in focus gene analysis. Genes shown are clustered according to the correlation of enriched signature/GO term membership. Correlations are illustrated in the heatmap on a *blue* (least) to *red* (most) scale as indicated at the top of the figure. Along the edges of the heatmap official gene symbols are provided. (PDF 1772 kb) Additional file 20: Figure S14. Relates to Fig. 9. IFN-responsive genes and the IFNγ-STAT1-IRF1 axis are amongst the leading edge of highly correlated DLBCL immune response genes. Correlation curves were generated from the focus gene analysis for all 16 genes used in the polarized immune response signature by ranking genes according to median correlation, and then plotting the gene correlation rank (x-axis) against the corresponding median gene correlation (y-axis, median Rho). This illustrates both the relative strength of correlations for each focus gene and identifies a leading edge of genes with most significant correlations. The position of a set of IFN-associated genes was plotted for each focus gene context as indicated in the figure. Note only the top 2000 of 20,121 genes tested are illustrated. (PDF 145 kb) Additional file 21: Figure S15. Relates to Fig. 10. Immune-modulatory and checkpoint gene expression is strongly correlated with elements of the polarized immune response signature. Correlation curves were generated from the focus gene analysis for all 16 genes used in the polarized immune response signature by ranking genes according to median correlation, and then plotting the gene correlation rank (x-axis) against the corresponding median gene correlation (y-axis, medianRHO). The position of immune checkpoint/modulatory genes on the resulting curves was plotted for each focus gene as indicated in the figure. Note only the top 2000 of 20,121 genes tested are illustrated. (PDF 140 kb)

## Abbreviations

ABC: activated B cell
CHOP: cyclophosphamide, doxorubicin hydrochloride (hydroxydaunomycin),vincristine sulfate (Oncovin), prednisone
COO: cell of origin
DLBCL: diffuse large B-cell lymphoma
EBV: Epstein-Barr virus
FDR: false discovery rate
GCB: germinal centre B cell
GEO: Gene Expression Omnibus
GO: gene ontology
HGNC: HUGO Gene Nomenclature Committee
IFN: interferon
NK: natural killer
PMBL: primary mediastinal B-cell lymphoma
R-CHOP: rituximab-CHOP
