# Supplementary material for: Gene expression meta-analysis reveals immune response convergence on the IFNγ-STAT1-IRF1 axis and adaptive immune resistance mechanisms in lymphoma
Source: Genome Med. 2015 Sep 11;7(1):96. doi: 10.1186/s13073-015-0218-3 (PMC4566848; doi:10.1186/s13073-015-0218-3)

Outline: Meta-profile generation

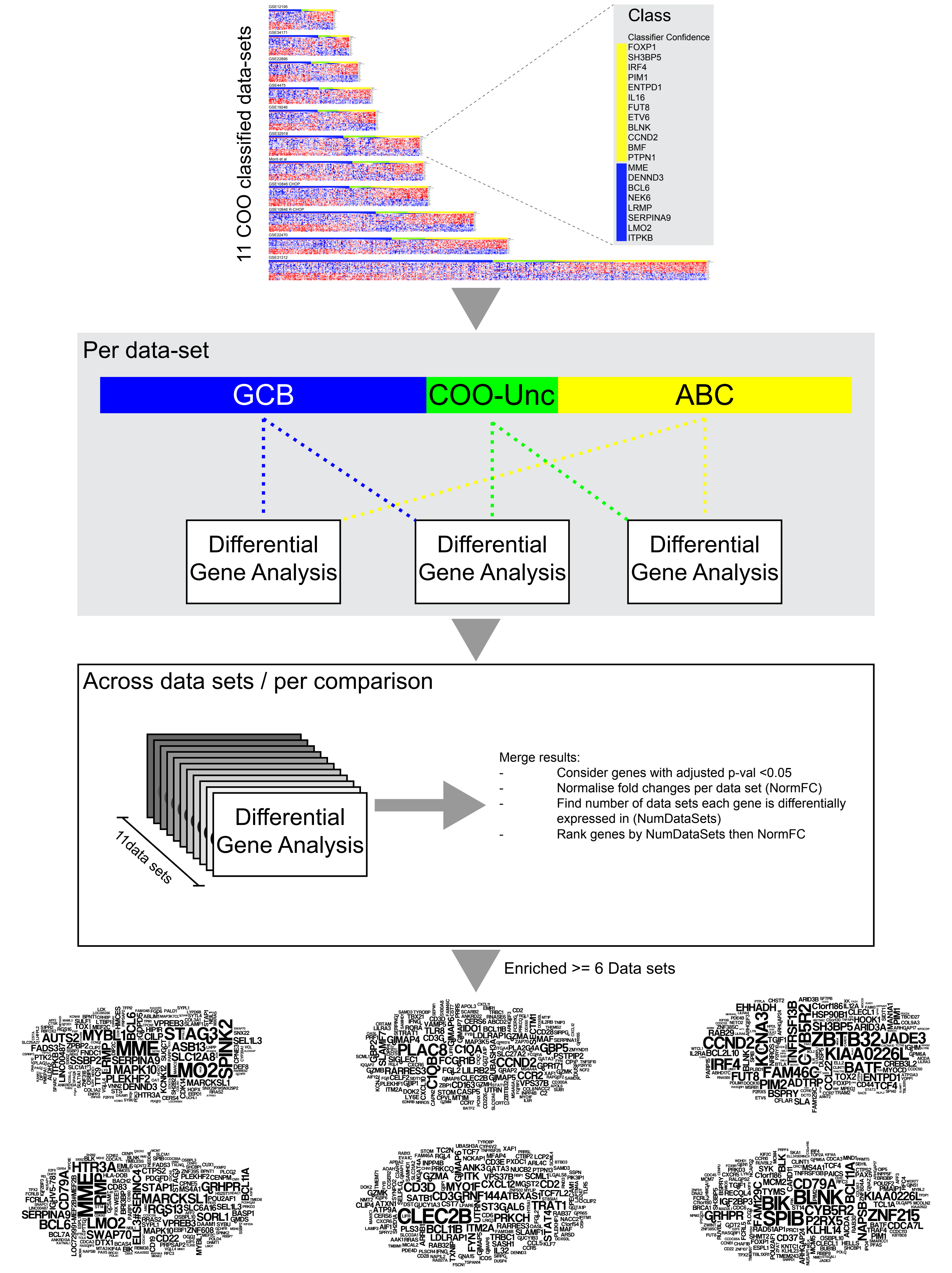

Supplement: Additional file 1: Figure S1. — Outline of meta-profile generation using COO classification. Upper panel: the data sets used. Please note one data set, GSE10846, is divided into two component parts reflecting underlying differences in treatment (CHOP versus R-CHOP), giving a total of 11 separate data set components. Illustrated are all cases for each data set, subdivided by COO classification established using the DAC classifier [32] and ranked by classification confidence, with classifier genes illustrated on the right (yellow bars ABC classifier genes and cases, blue bars GCB classifier genes and cases, green bars COO-unclassified cases). Middle panels: the pairwise comparisons of differentially expressed genes between three classes, and the integration of differentially expressed genes across data sets. Bottom panels: the resulting meta-profiles of differentially expressed genes for both components of the three possible pairwise comparisons are shown as Wordles for illustrative purposes (complete details provided in Additional file 5). (PDF 2024 kb) [file 13073_2015_218_MOESM1_ESM.pdf]
