# Supplementary figures and images for: Gene expression meta-analysis reveals immune response convergence on the IFNγ-STAT1-IRF1 axis and adaptive immune resistance mechanisms in lymphoma
Source: Genome Med. 2015 Sep 11;7(1):96. doi: 10.1186/s13073-015-0218-3 (PMC4566848; doi:10.1186/s13073-015-0218-3)

# Outline: Signature enrichment visualisation

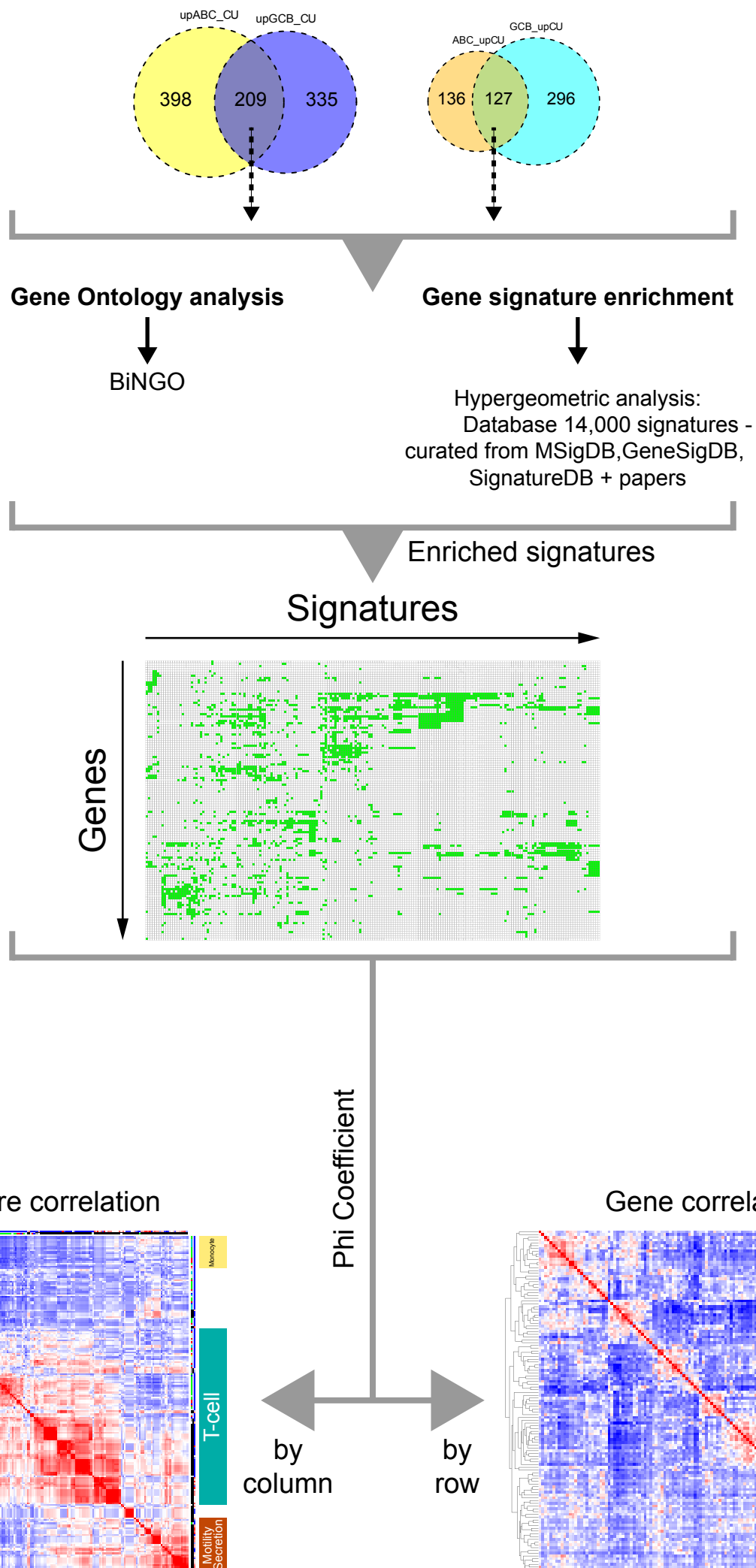

Supplement: Additional file 2: Figure S2. — Outline of the process for integrating and visualizing analysis of gene signature and ontology enrichments. The flow diagram illustrates the process for integrating gene signature and ontology enrichments. The initial assessment of overlap between meta-profiles derived from the comparison of ABC-DLBCL versus COO-unclassified (CU) DLBCL and GCB-DLBCL versus COO-unclassified (CU) DLBCL is shown at the top of the figure, followed by the parallel analysis of gene ontology (BiNGO) and hypergeometric testing of signature enrichments. Next a matrix is illustrated showing the occurrence of genes versus enriched signatures (green fill), followed by analysis of correlations (Phi coefficient) by column (signature/ontology terms) or by row (genes) and hierarchical clustering. (PDF 1658 kb) [file 13073_2015_218_MOESM2_ESM.pdf]

Outline: Focus gene analysis

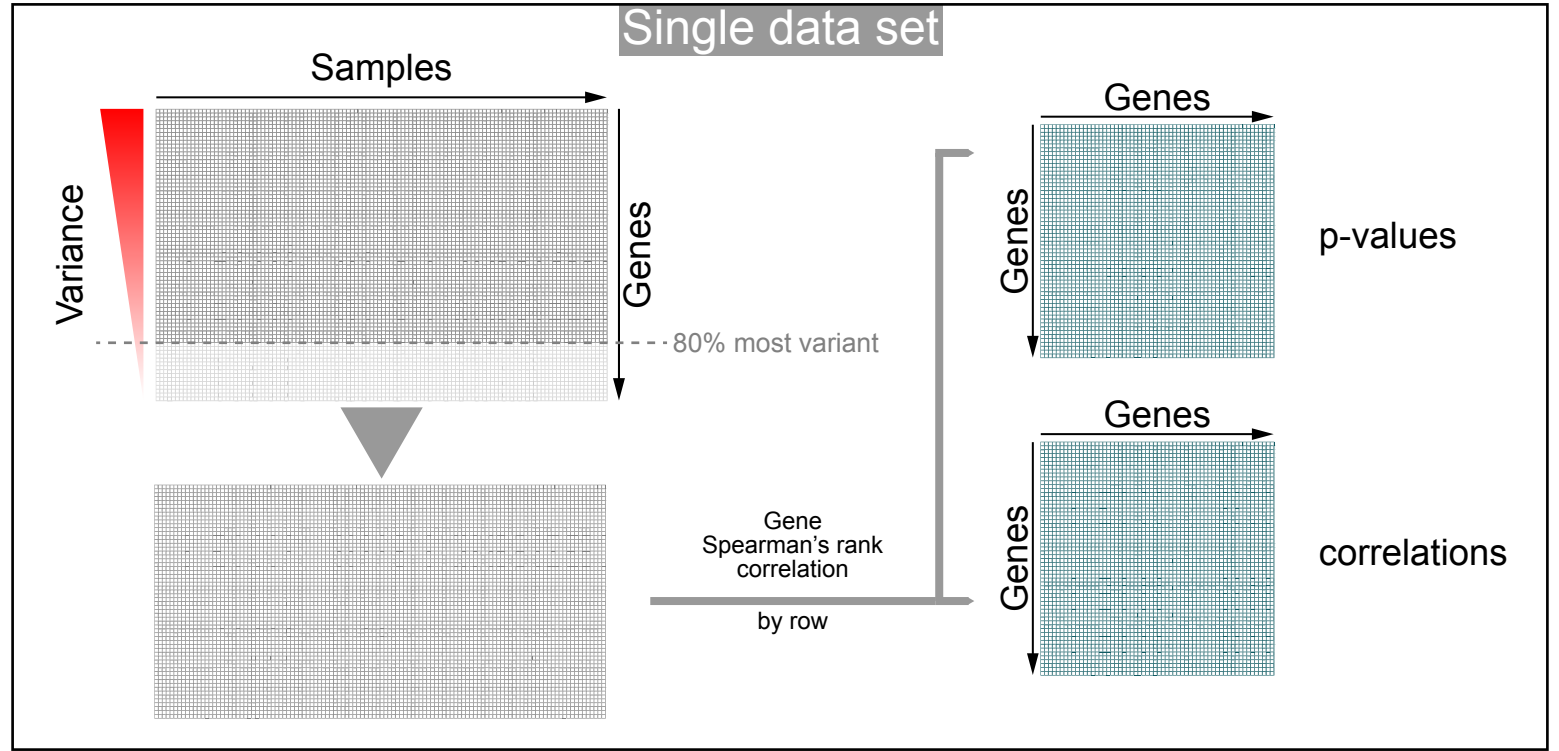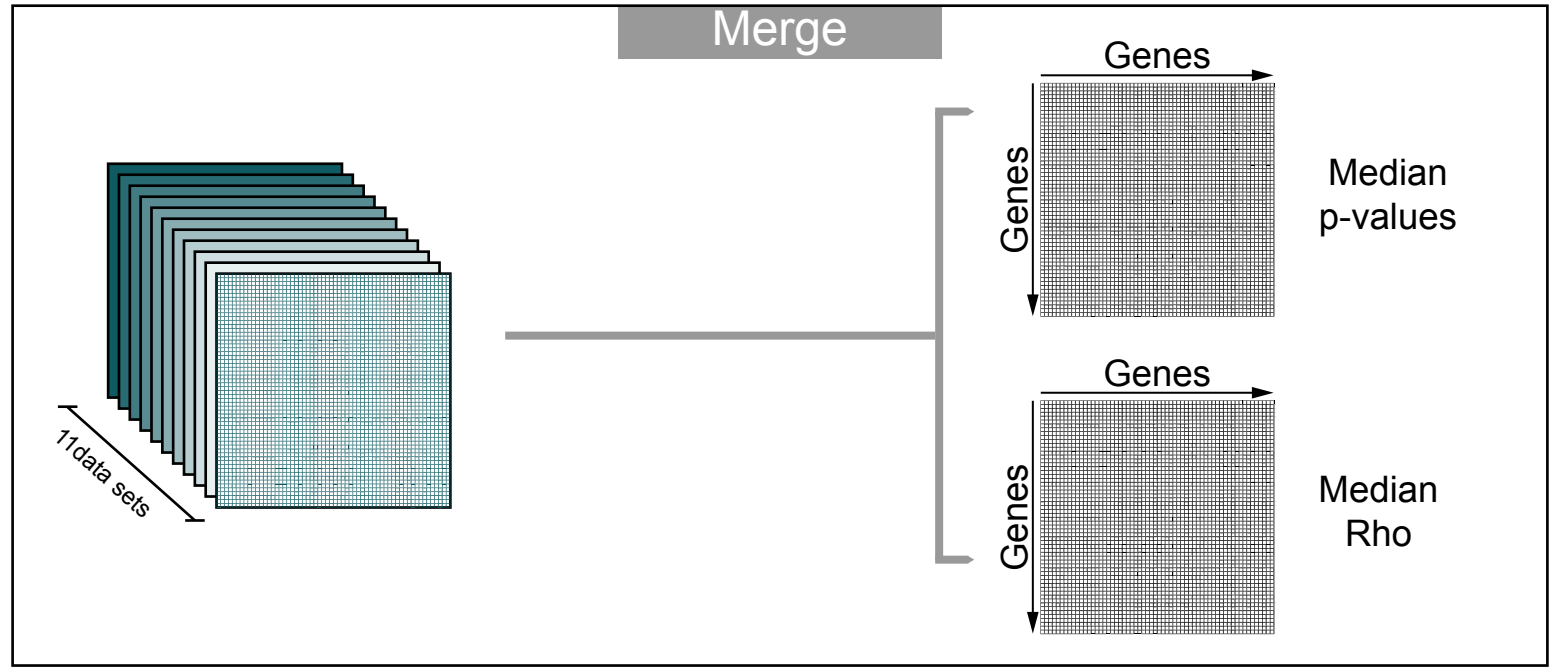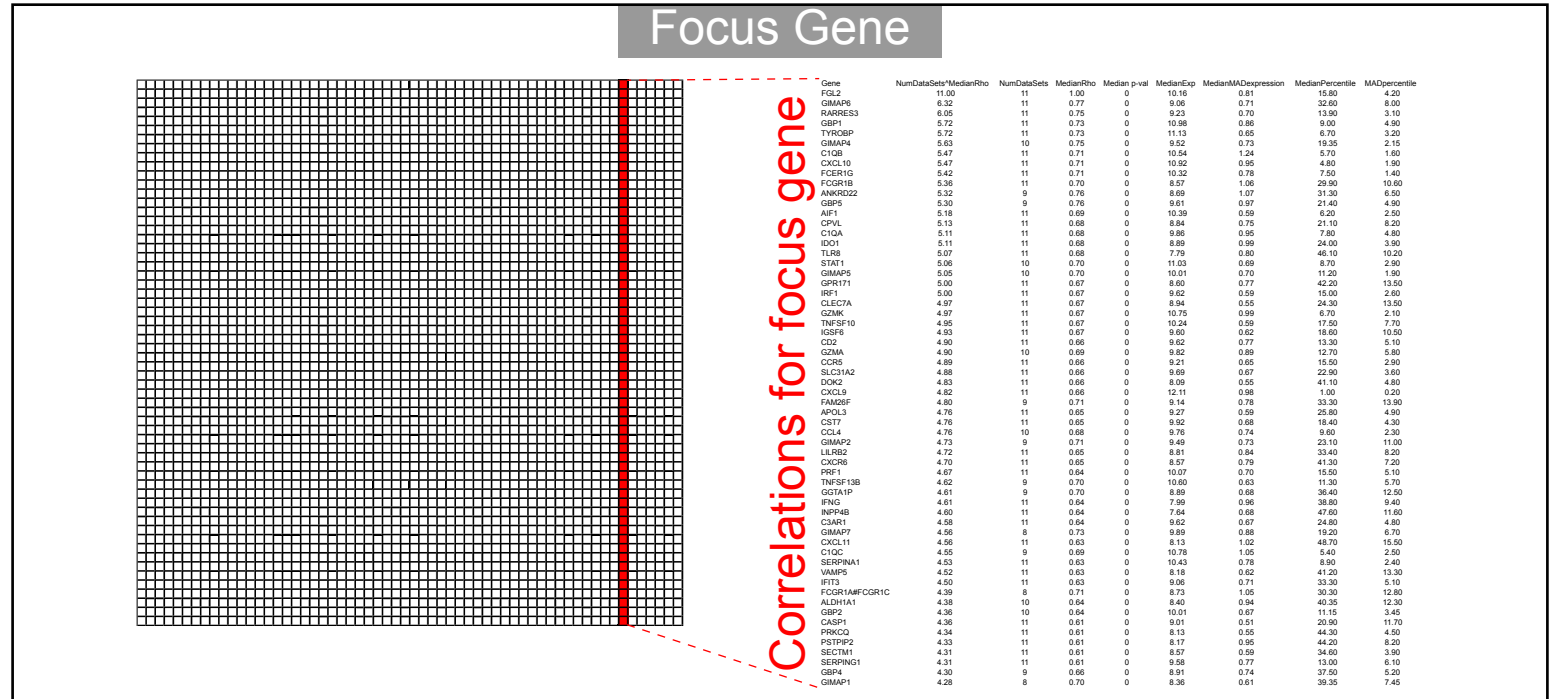

Supplement: Additional file 3: Figure S11. — An outline of the focus gene approach, and a high resolution image to accompany Fig. 8a. Upper panel: the approach within each data set with initial selection of the 80 % most variable genes, and subsequent generation of linked matrices of gene correlation values and associated p values. Middle panel: merging of all data sets (11 data sets; data set GSE10846 subdivided by treatment type) to give gene by gene matrices of median correlations and p values. Lower panel: the selection of an individual focus gene for downstream analysis. (PDF 1375 kb) [file 13073_2015_218_MOESM3_ESM.pdf]

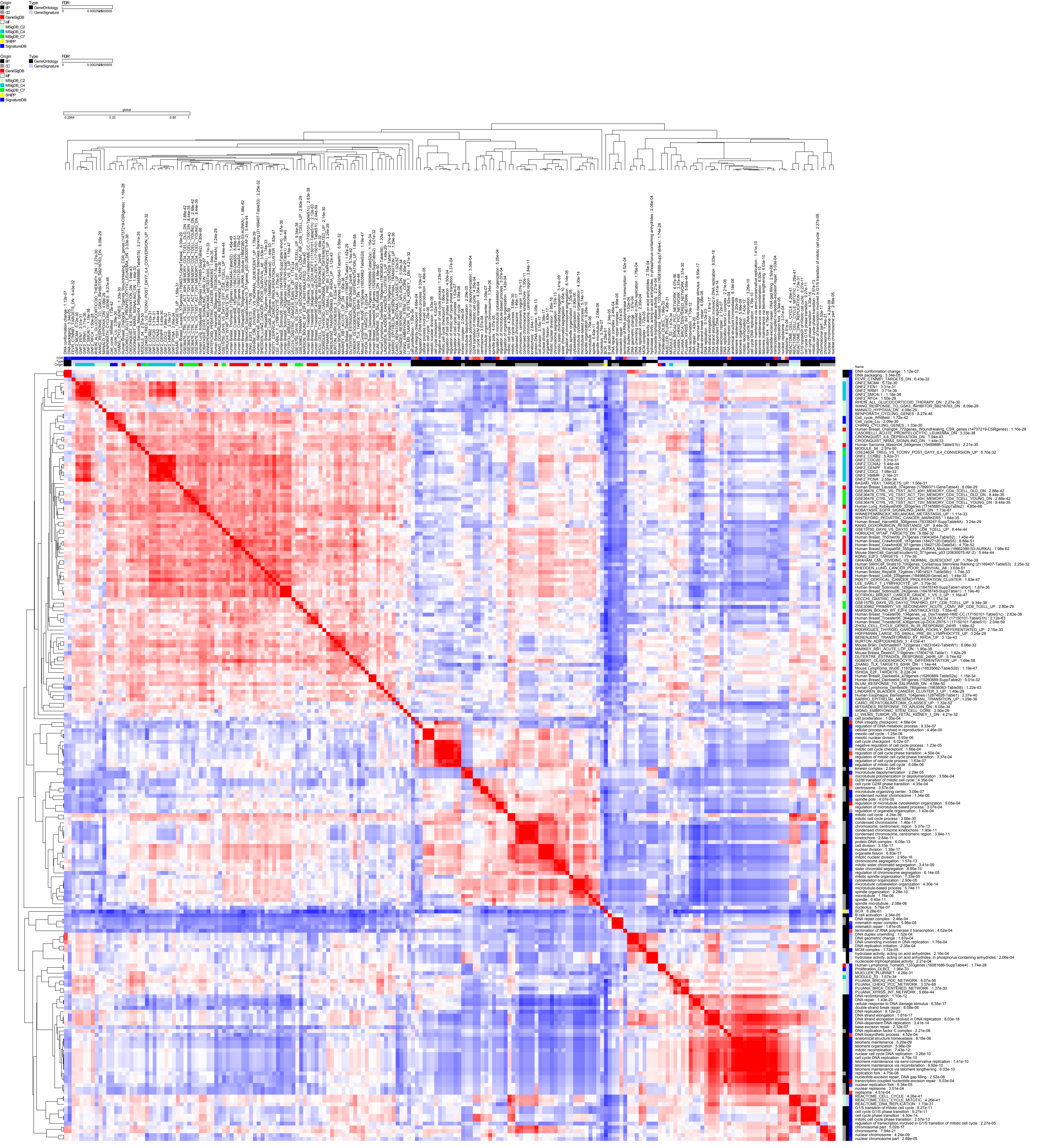

Supplement: Additional file 7: Figure S3. — High resolution image corresponding to Fig. 2a. Integrated gene signature and ontology enrichments for COO-classified meta-profile clusters from signature and ontology term perspectives. The figure represents the hierarchical clustering of enriched gene signature and ontology terms related to the COO-classified meta-profile. Correlations are illustrated in heat maps on a blue (least) to red (most) scale as indicated at the top of the figure. Along the edges of the heatmap the signature terms are provided (and correspond to terms listed in Additional file 6). The FDR-corrected p value for enrichment of the signature is illustrated as a bar on either side of the heatmap, along with an indication of the type of term (signature versus ontology) and the origin of the terms as indicated in the figure. (PDF 283 kb) [file 13073_2015_218_MOESM7_ESM.pdf]

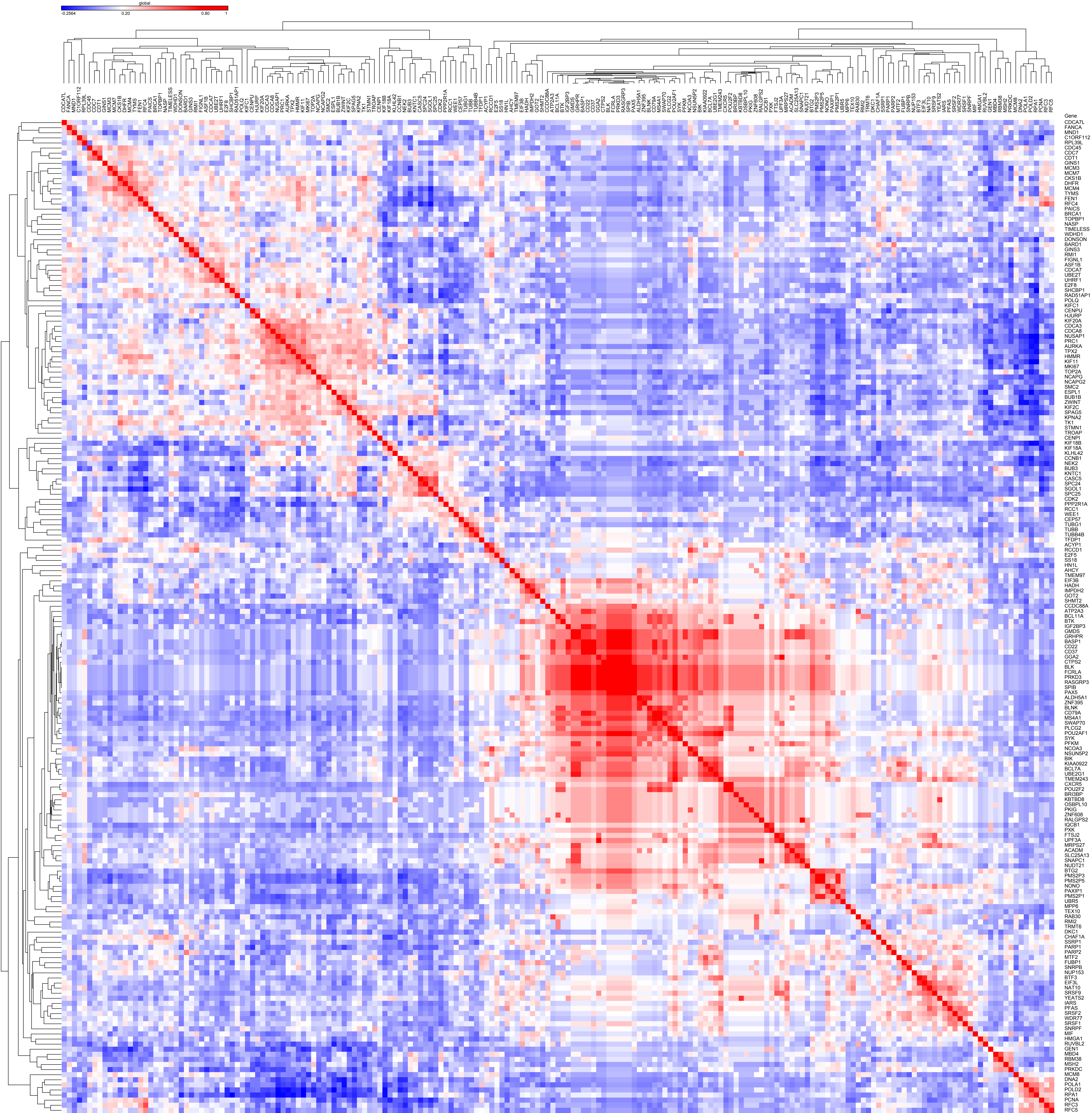

Supplement: Additional file 8: Figure S4. — High resolution image corresponding to Fig. 2b. Integrated gene signature and ontology enrichments for COO-classified meta-profile clustered from the gene perspective. The figure represents the hierarchical clustering of meta-profile genes contributing to signature and ontology term enrichments, and clustered according to the correlation of enriched signature/ontology term membership. Correlations are illustrated in the heatmap on a blue (least) to red (most) scale as indicated at the top of the figure. Along the edges of the heatmap official gene symbols are provided. (PDF 251 kb) [file 13073_2015_218_MOESM8_ESM.pdf]

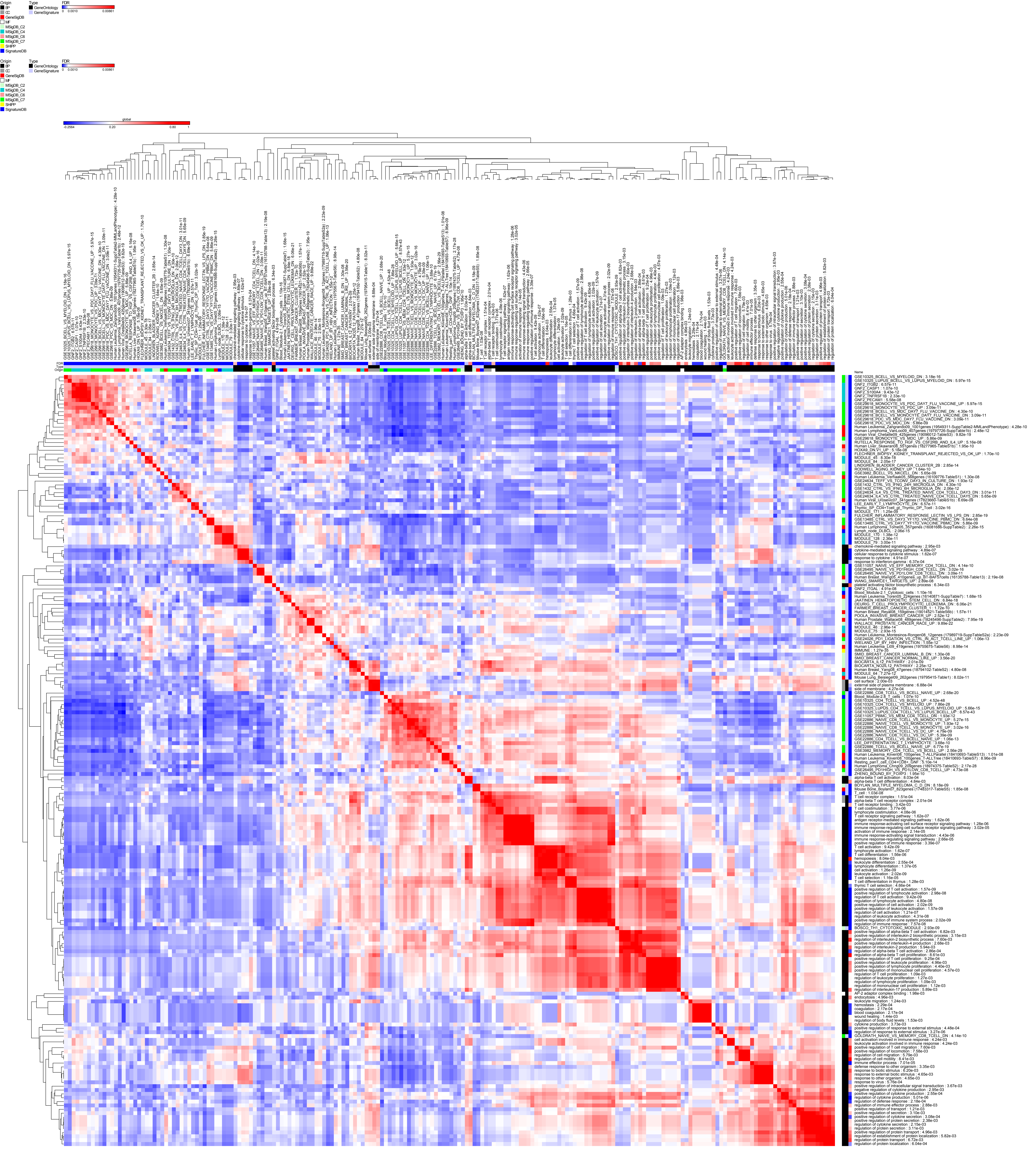

Supplement: Additional file 9: Figure S5. — High resolution image corresponding to Fig. 3a. Integrated gene signature and ontology enrichments for COO-unclassified meta-profile clusters from signature and ontology term perspectives. The figure represents the hierarchical clustering of enriched gene signature and ontology terms related to the COO-unclassified meta-profile. Correlations are illustrated in heat maps on a blue (least) to red (most) scale as indicated at the top of the figure. Along the edges of the heatmap the signature terms are provided (and correspond to terms listed in Additional file 6). The FDR-corrected p value for enrichment of the signature is illustrated as a bar on either side of the heatmap, along with an indication of the type of term (signature versus ontology) and the origin of the terms as indicated in the figure. (PDF 518 kb) [file 13073_2015_218_MOESM9_ESM.pdf]

## Signature Correlation

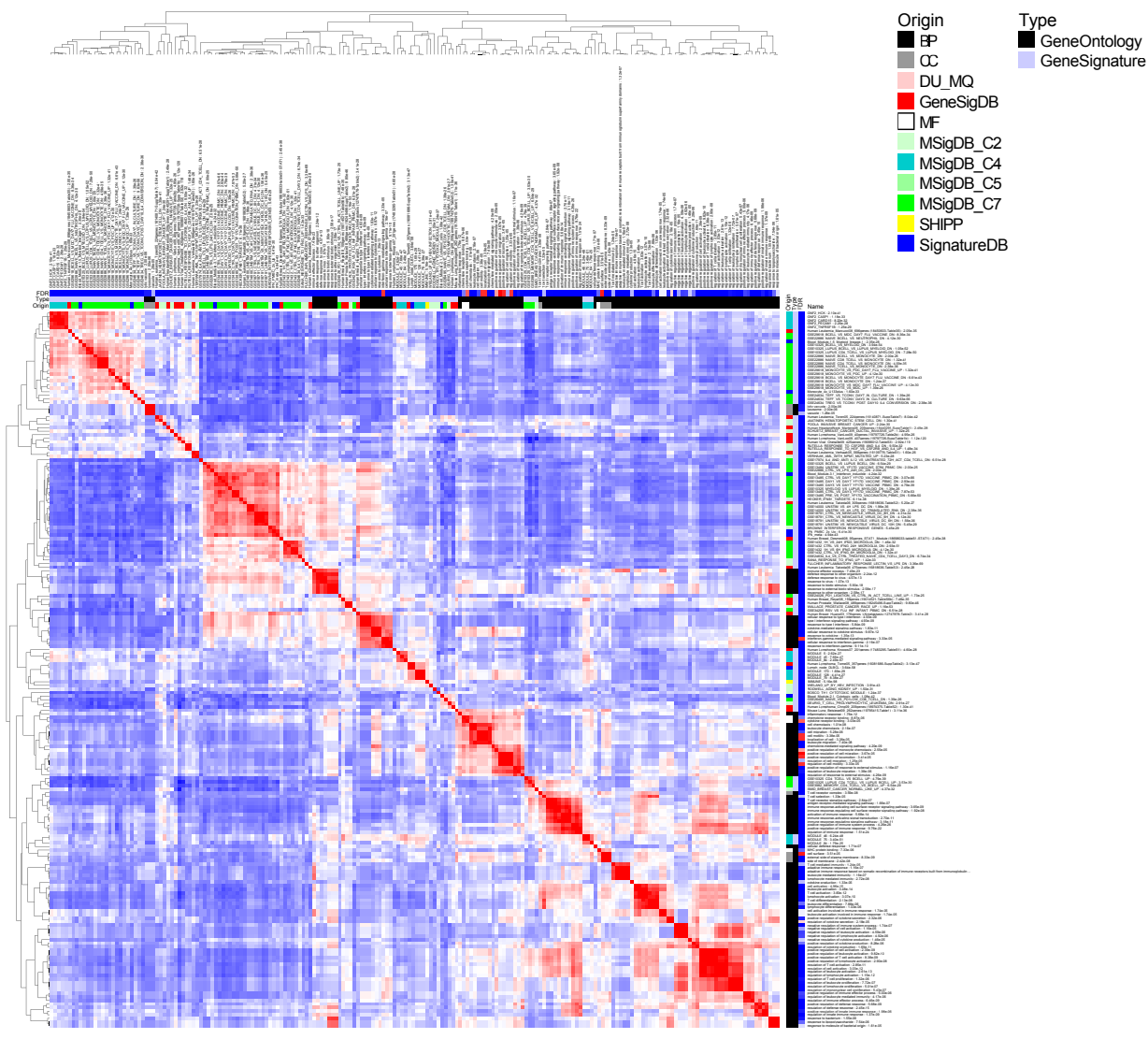

## Gene Correlation

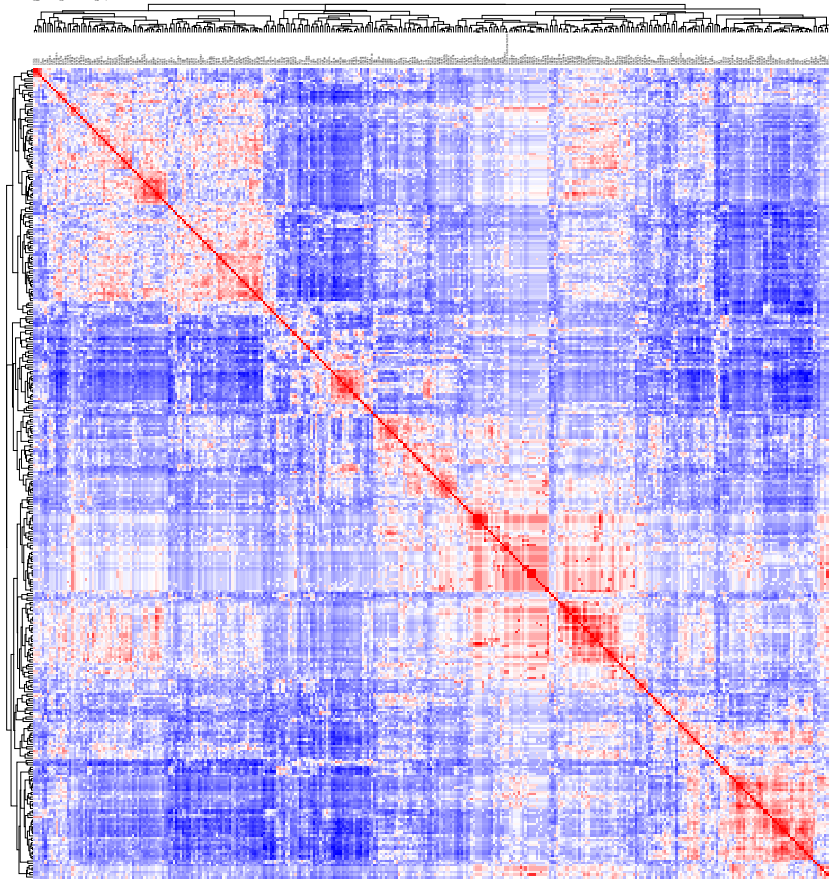

Supplement: Additional file 19: Figure S13. — Relates to Fig. 8b. Integrated gene signature and ontology enrichments for FGL2 focus gene analysis. Upper panel: the hierarchical clustering of enriched gene signature and GO terms related to genes correlating with FGL2 in focus gene analysis. Correlations are illustrated in the heatmap on a blue (least) to red (most) scale as indicated at the top of the figure. Along the edges of the heatmap the signature terms are provided (and correspond to terms listed in Additional file 17). The FDR-corrected p value for enrichment of the signature is illustrated as a bar on either side of the heatmap, along with an indication of the type of term (signature versus ontology) and the origin of the terms as indicated in the figure. Lower panel: the hierarchical clustering of genes correlating with FGL2 in focus gene analysis. Genes shown are clustered according to the correlation of enriched signature/GO term membership. Correlations are illustrated in the heatmap on a blue (least) to red (most) scale as indicated at the top of the figure. Along the edges of the heatmap official gene symbols are provided. (PDF 1772 kb) [file 13073_2015_218_MOESM19_ESM.pdf]

**BCL11B**

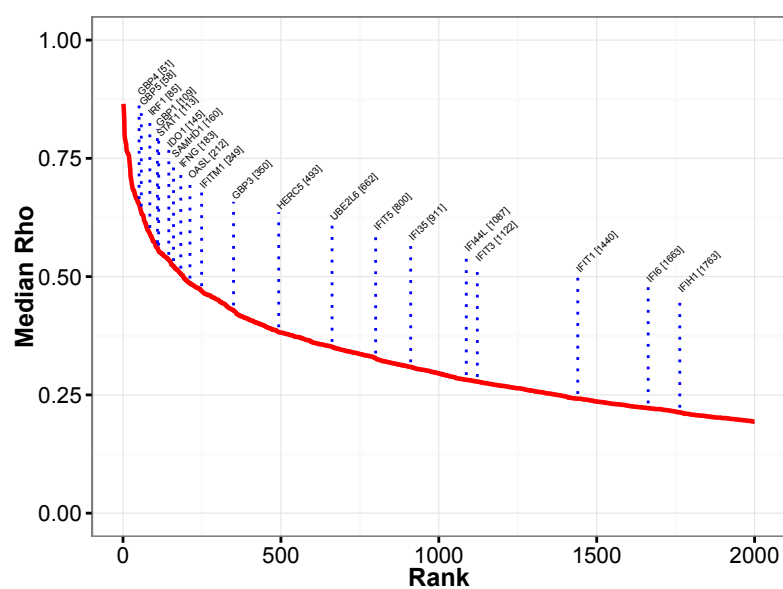

CD2

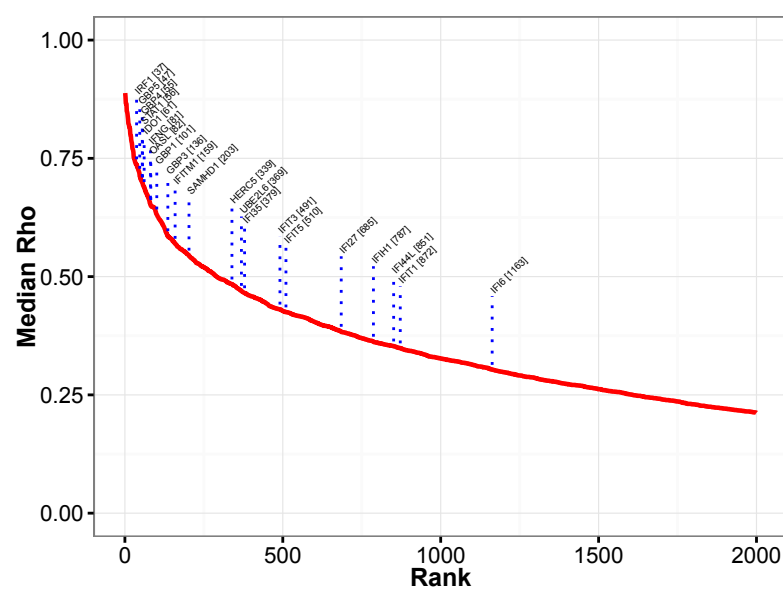

CD3D

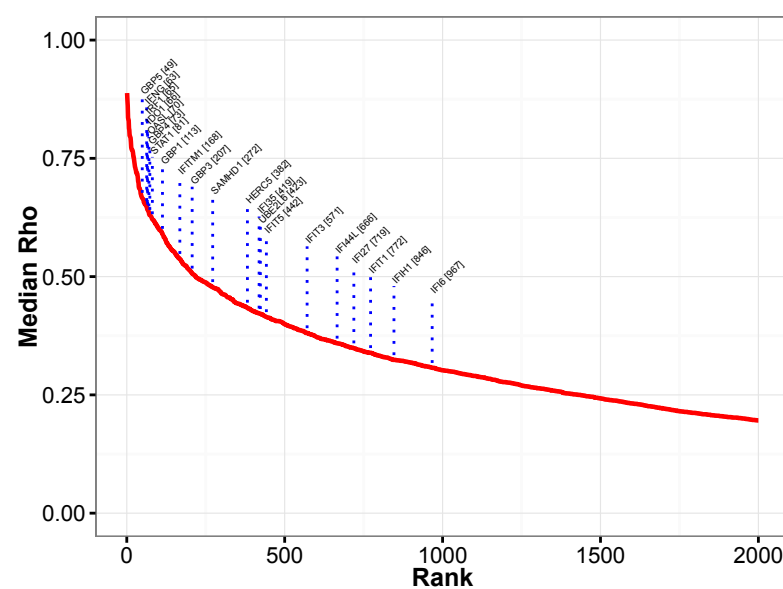

CD3G

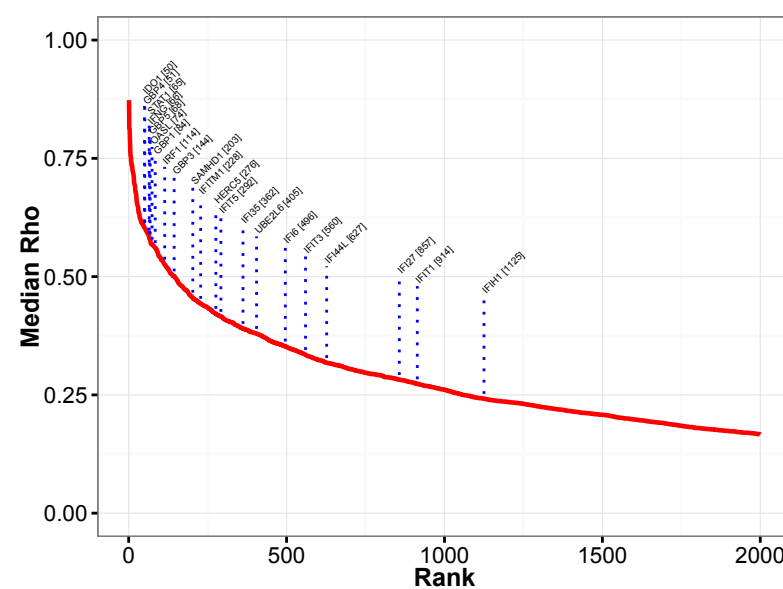

# CLEC2B

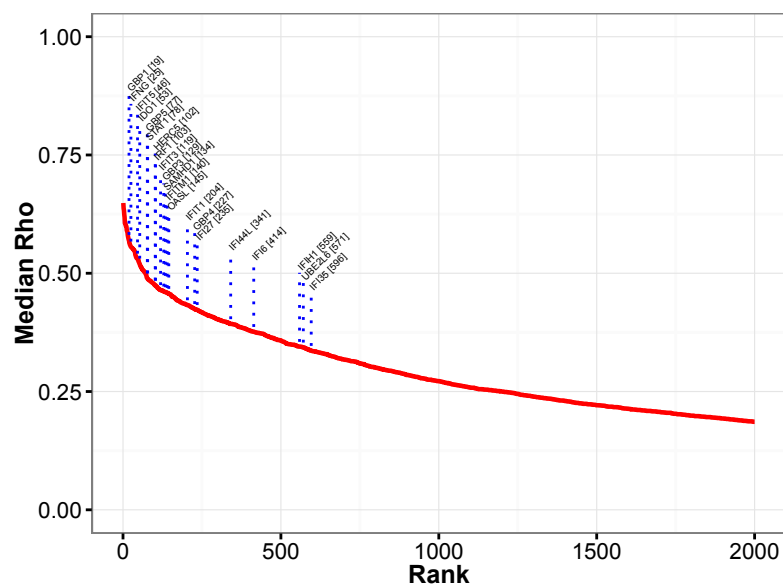

FGL2

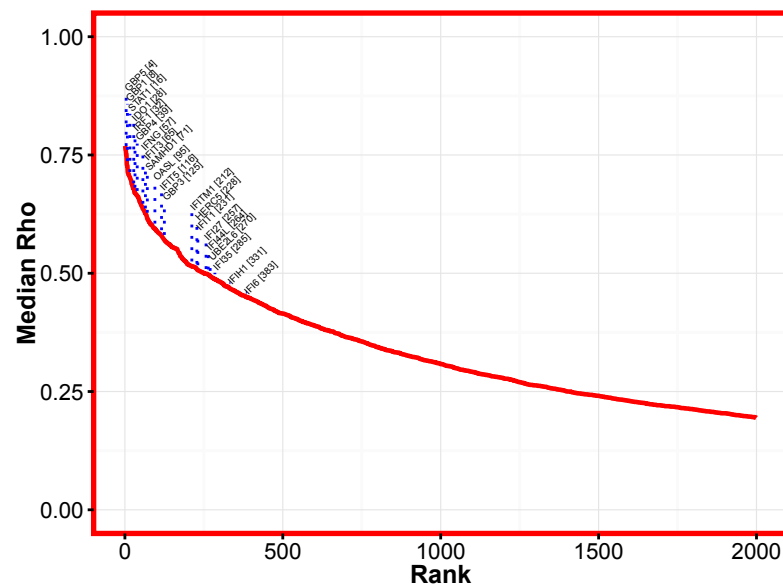

# GIMAP6

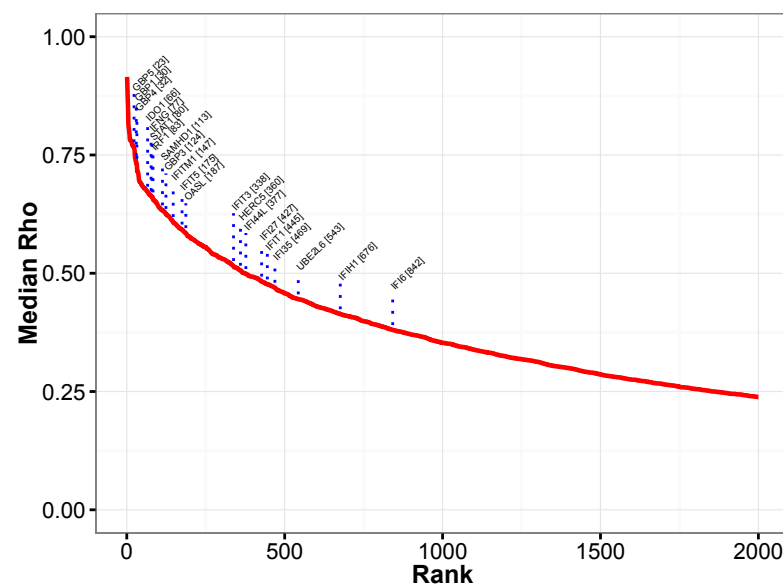

GZMA

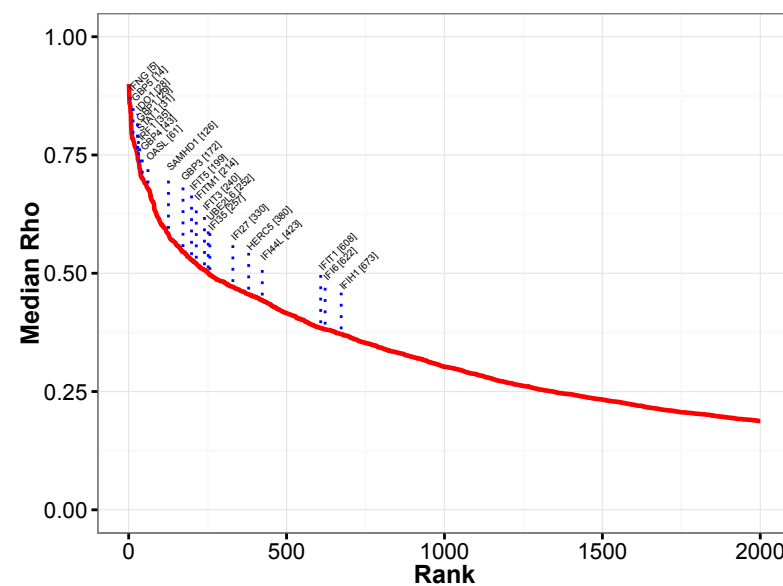

GZMK

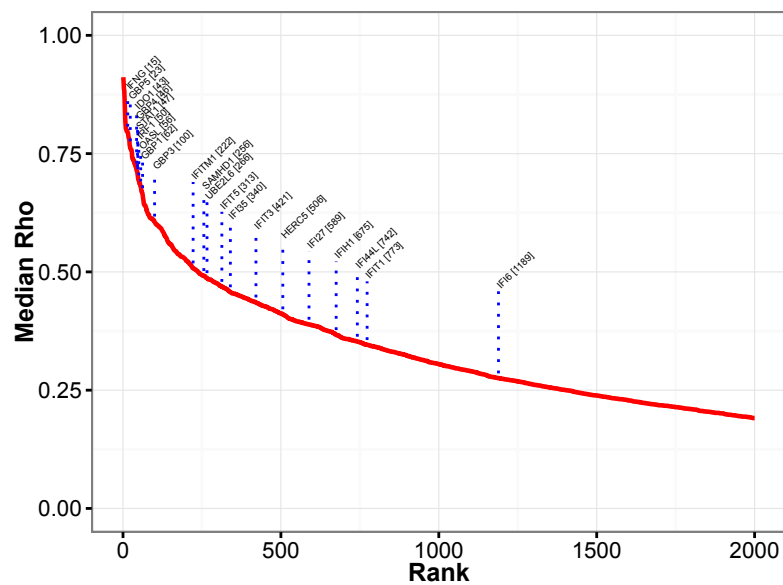

IFNG

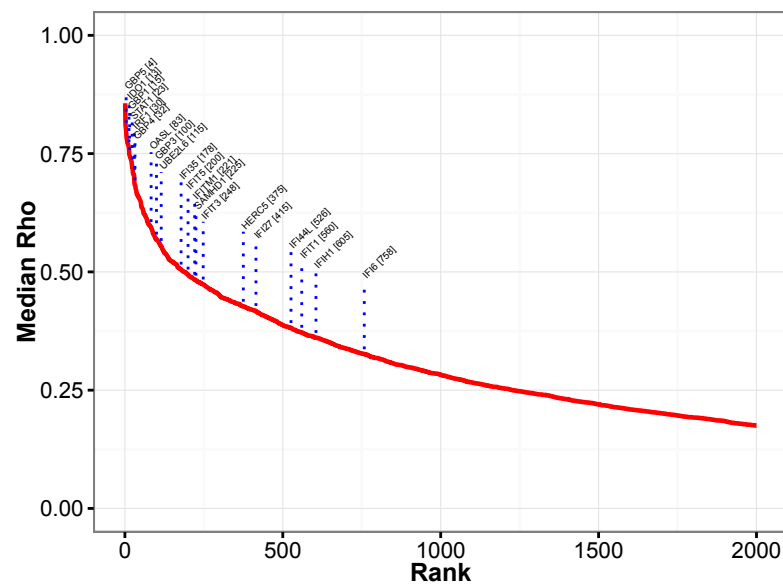

ITM2A

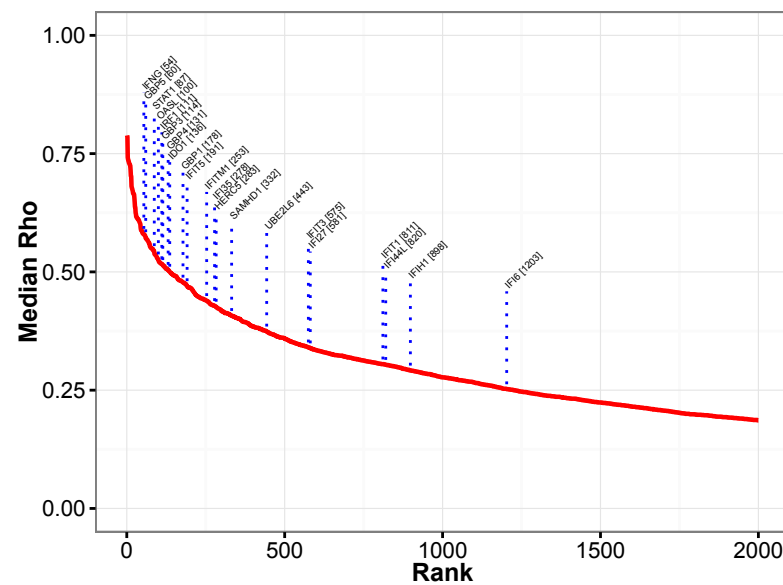

RARRES3

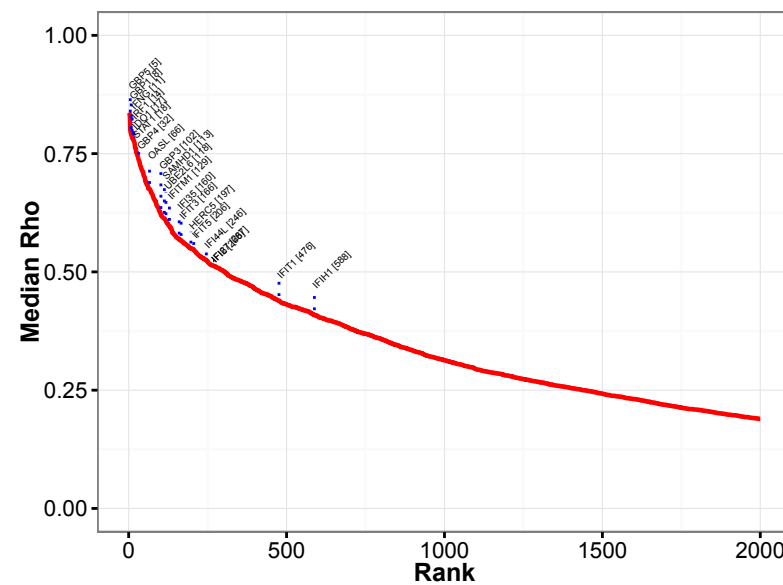

TCN2

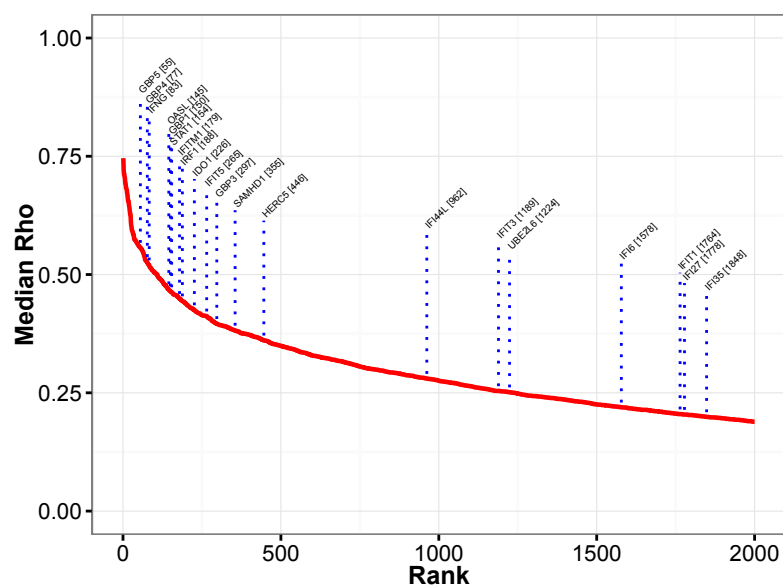

TRAT1

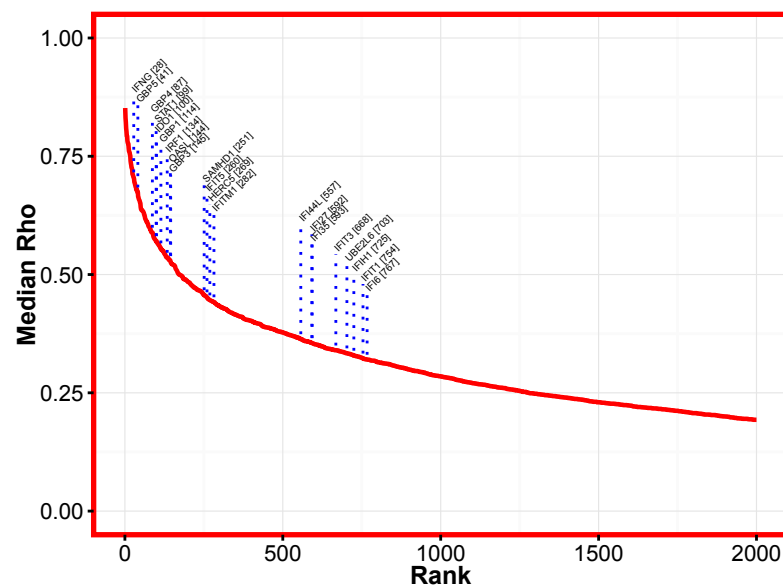

TRBC1

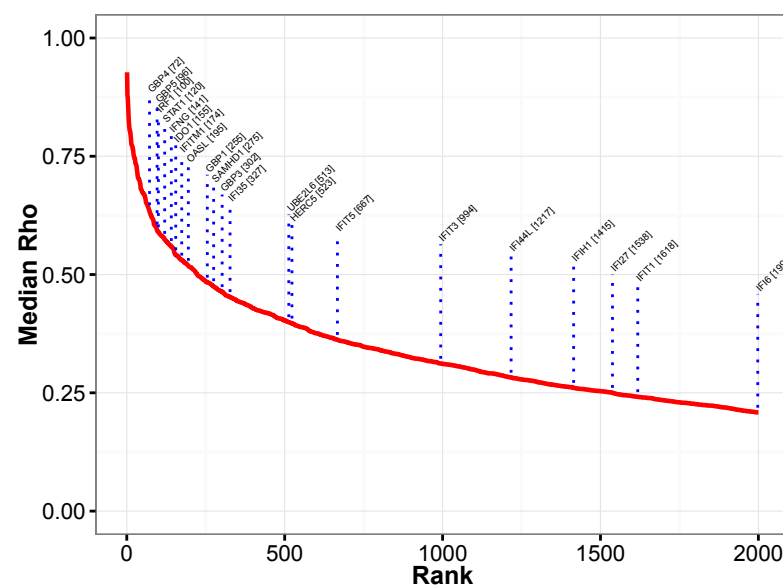

UBASH3A

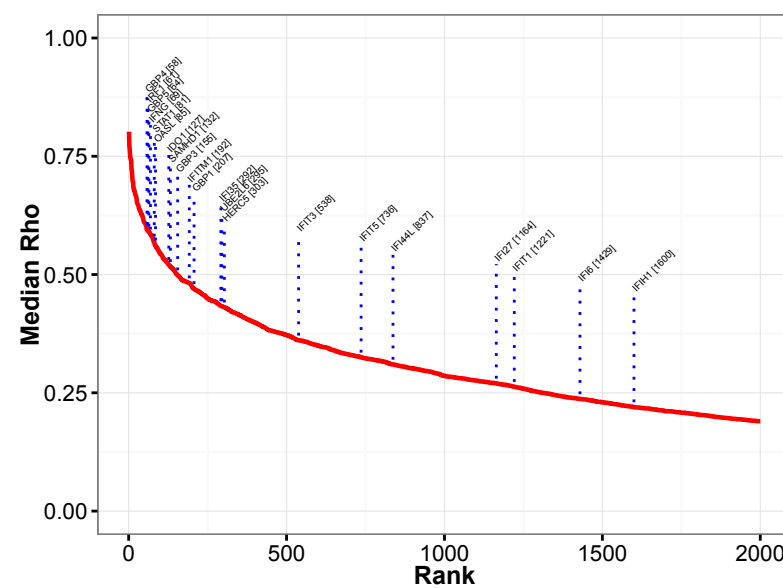

Supplement: Additional file 20: Figure S14. — Relates to Fig. 9. IFN-responsive genes and the IFNγ-STAT1-IRF1 axis are amongst the leading edge of highly correlated DLBCL immune response genes. Correlation curves were generated from the focus gene analysis for all 16 genes used in the polarized immune response signature by ranking genes according to median correlation, and then plotting the gene correlation rank (x-axis) against the corresponding median gene correlation (y-axis, median Rho). This illustrates both the relative strength of correlations for each focus gene and identifies a leading edge of genes with most significant correlations. The position of a set of IFN-associated genes was plotted for each focus gene context as indicated in the figure. Note only the top 2000 of 20,121 genes tested are illustrated. (PDF 145 kb) [file 13073_2015_218_MOESM20_ESM.pdf]

BCL11B

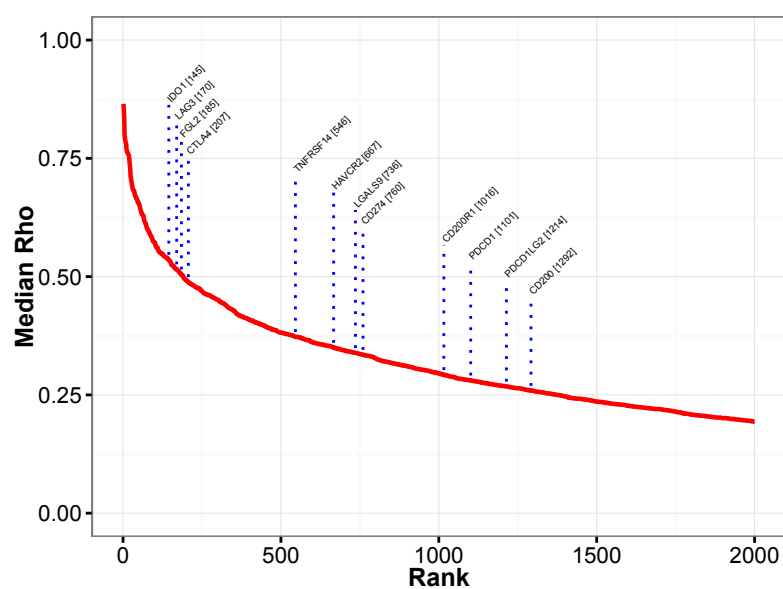

CD2

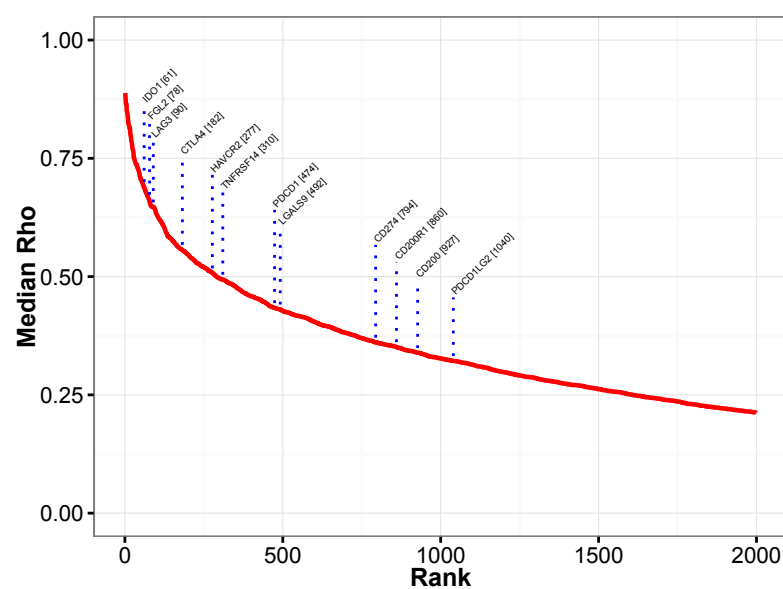

CD3D

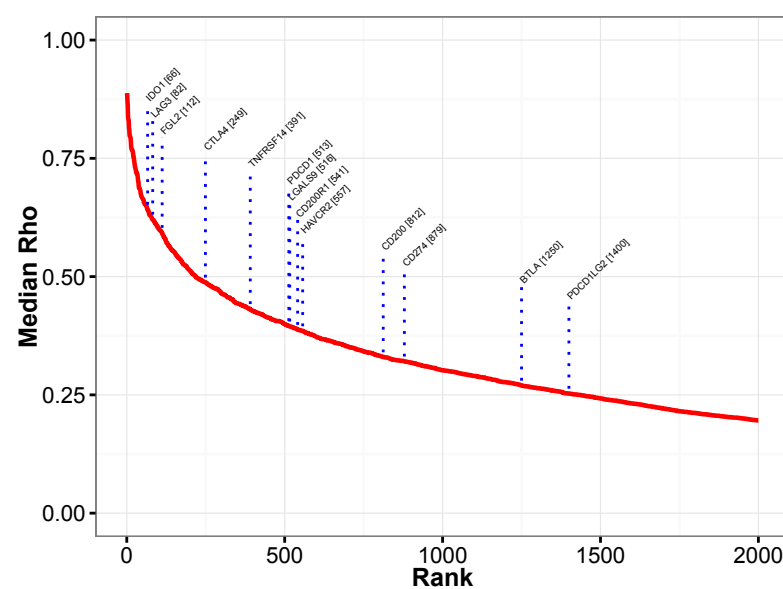

CD3G

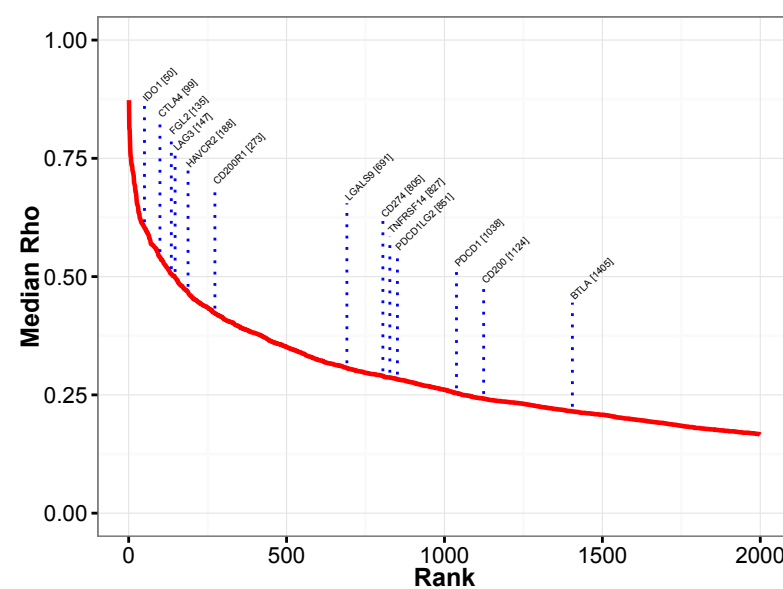

CLEC2B

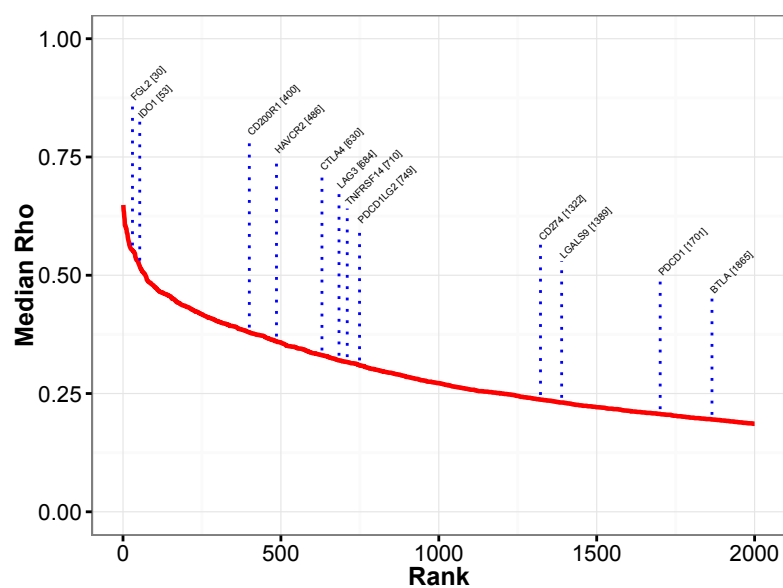

FGL2

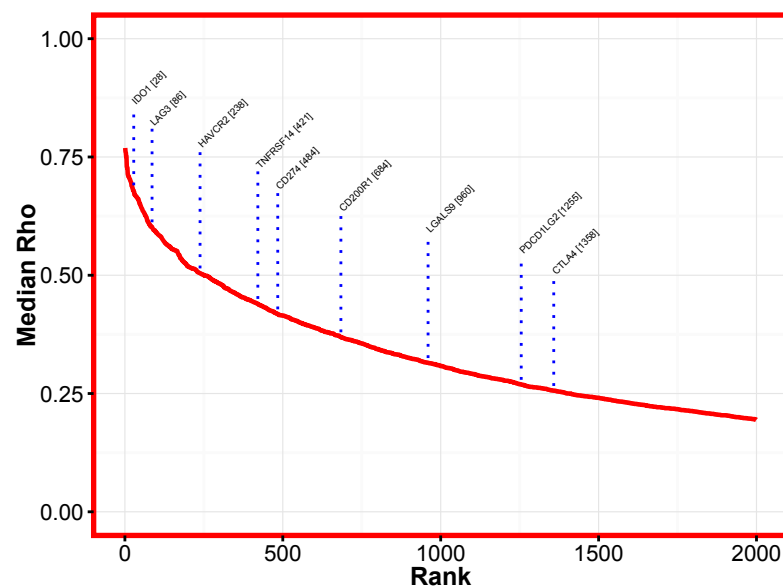

GIMAP6

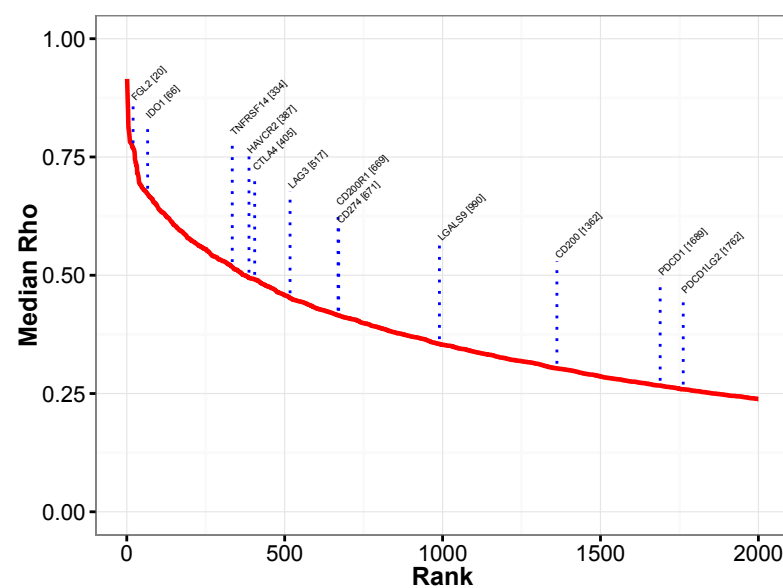

GZMA

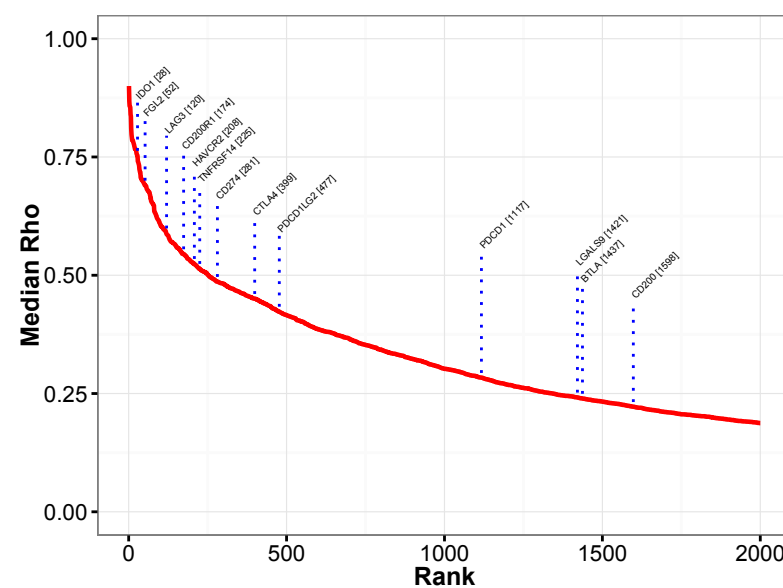

GZMK

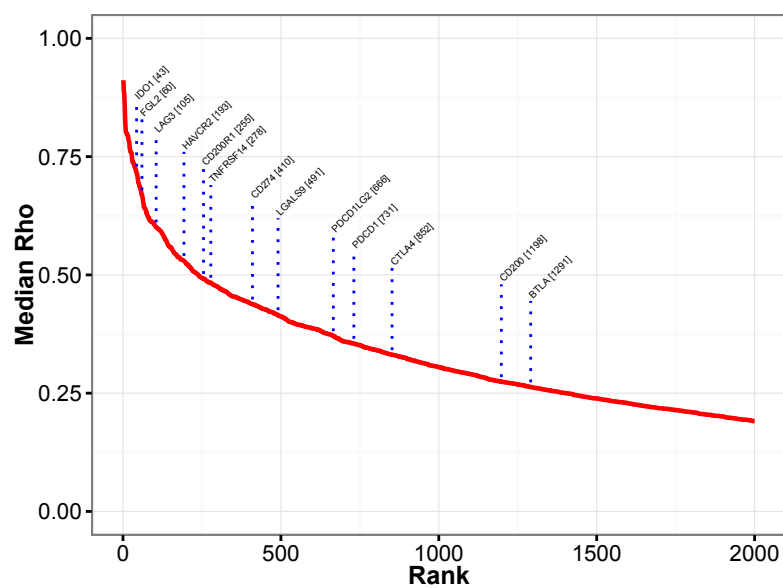

IFNG

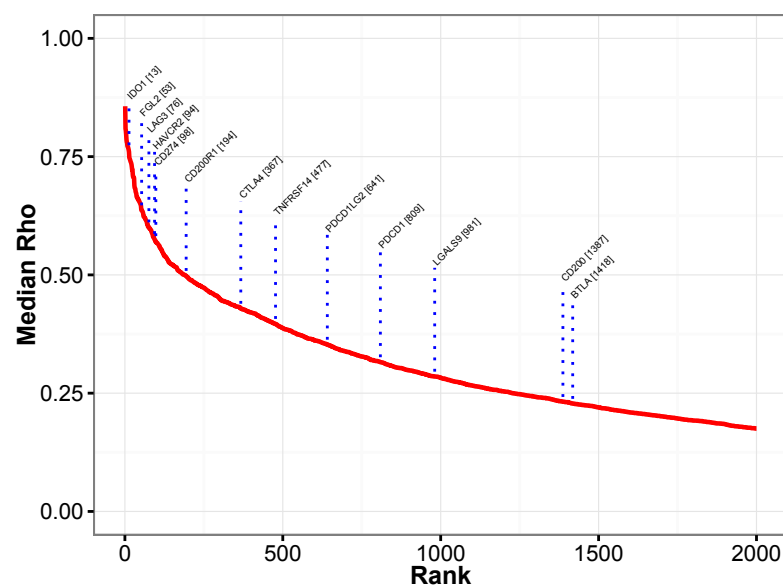

ITM2A

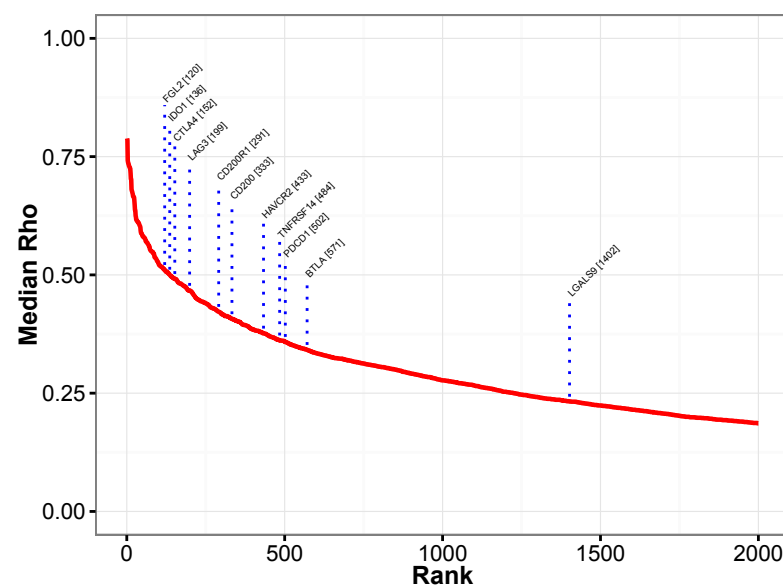

RARRES3

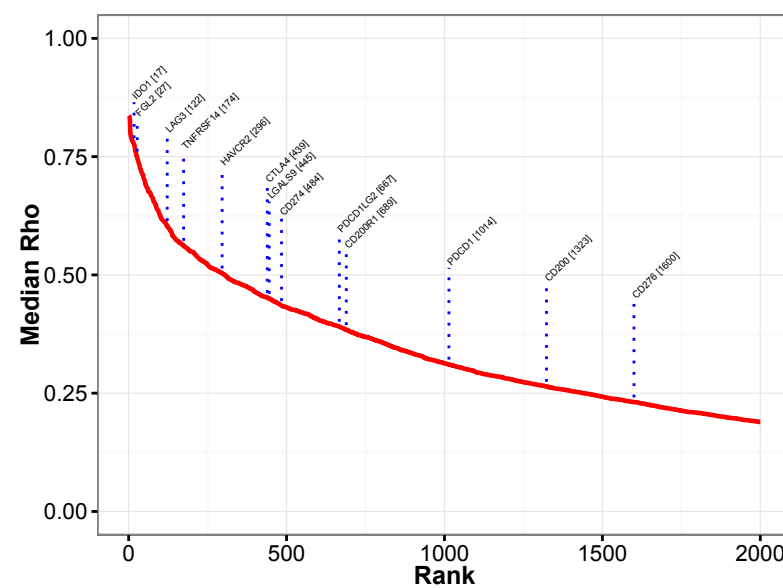

TCN2

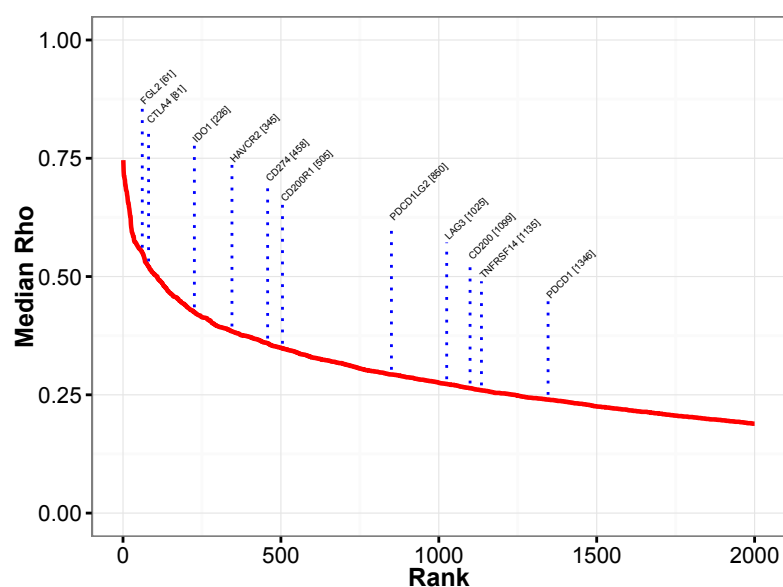

TRAT1

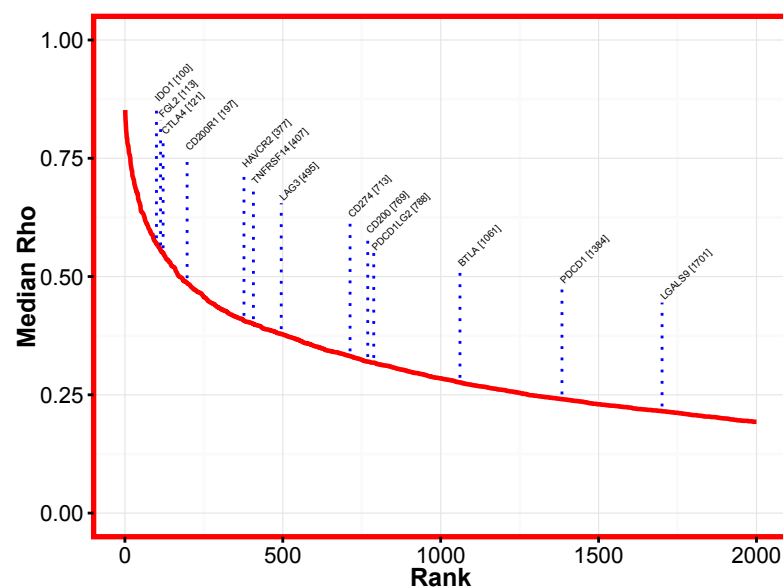

TRBC1

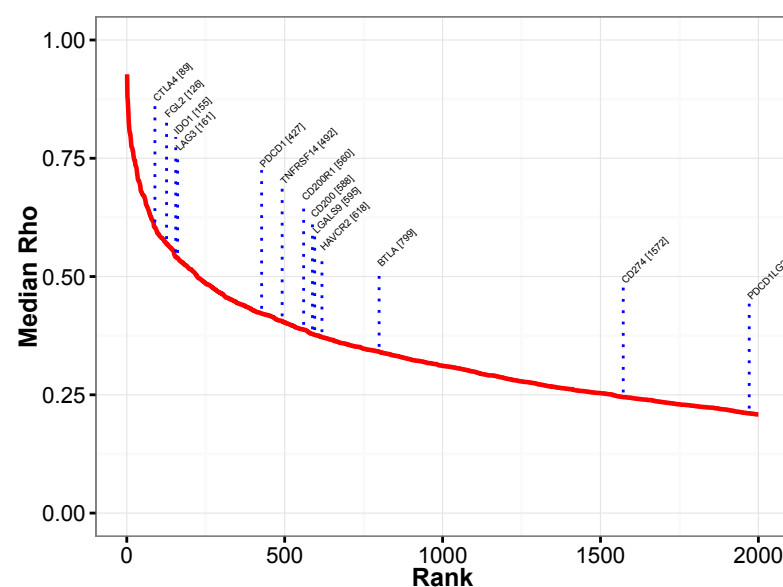

UBASH3A

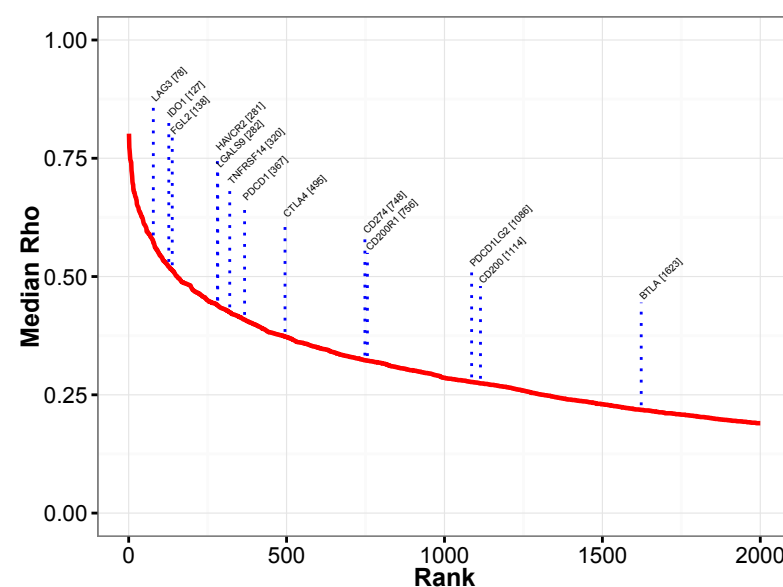

Supplement: Additional file 21: Figure S15. — Relates to Fig. 10. Immune-modulatory and checkpoint gene expression is strongly correlated with elements of the polarized immune response signature. Correlation curves were generated from the focus gene analysis for all 16 genes used in the polarized immune response signature by ranking genes according to median correlation, and then plotting the gene correlation rank (x-axis) against the corresponding median gene correlation (y-axis, medianRHO). The position of immune checkpoint/modulatory genes on the resulting curves was plotted for each focus gene as indicated in the figure. Note only the top 2000 of 20,121 genes tested are illustrated. (PDF 140 kb) [file 13073_2015_218_MOESM21_ESM.pdf]
