# Supplementary material for: Gene expression meta-analysis reveals immune response convergence on the IFNγ-STAT1-IRF1 axis and adaptive immune resistance mechanisms in lymphoma
Source: Genome Med. 2015 Sep 11;7(1):96. doi: 10.1186/s13073-015-0218-3 (PMC4566848; doi:10.1186/s13073-015-0218-3)
Supplement: Additional file 10: Figure S6. — High resolution image corresponding to Fig. 3b. Integrated gene signature and ontology enrichments for COO-unclassified meta-profile clustered from the gene perspective. The figure represents the hierarchical clustering of meta-profile genes contributing to signature and ontology term enrichments, and clustered according to the correlation of enriched signature/ontology term membership. Correlations are illustrated in the heatmap on a blue (least) to red (most) scale as indicated at the top of the figure. Along the edges of the heatmap official gene symbols are provided. (PDF 126 kb) [file 13073_2015_218_MOESM10_ESM.pdf]

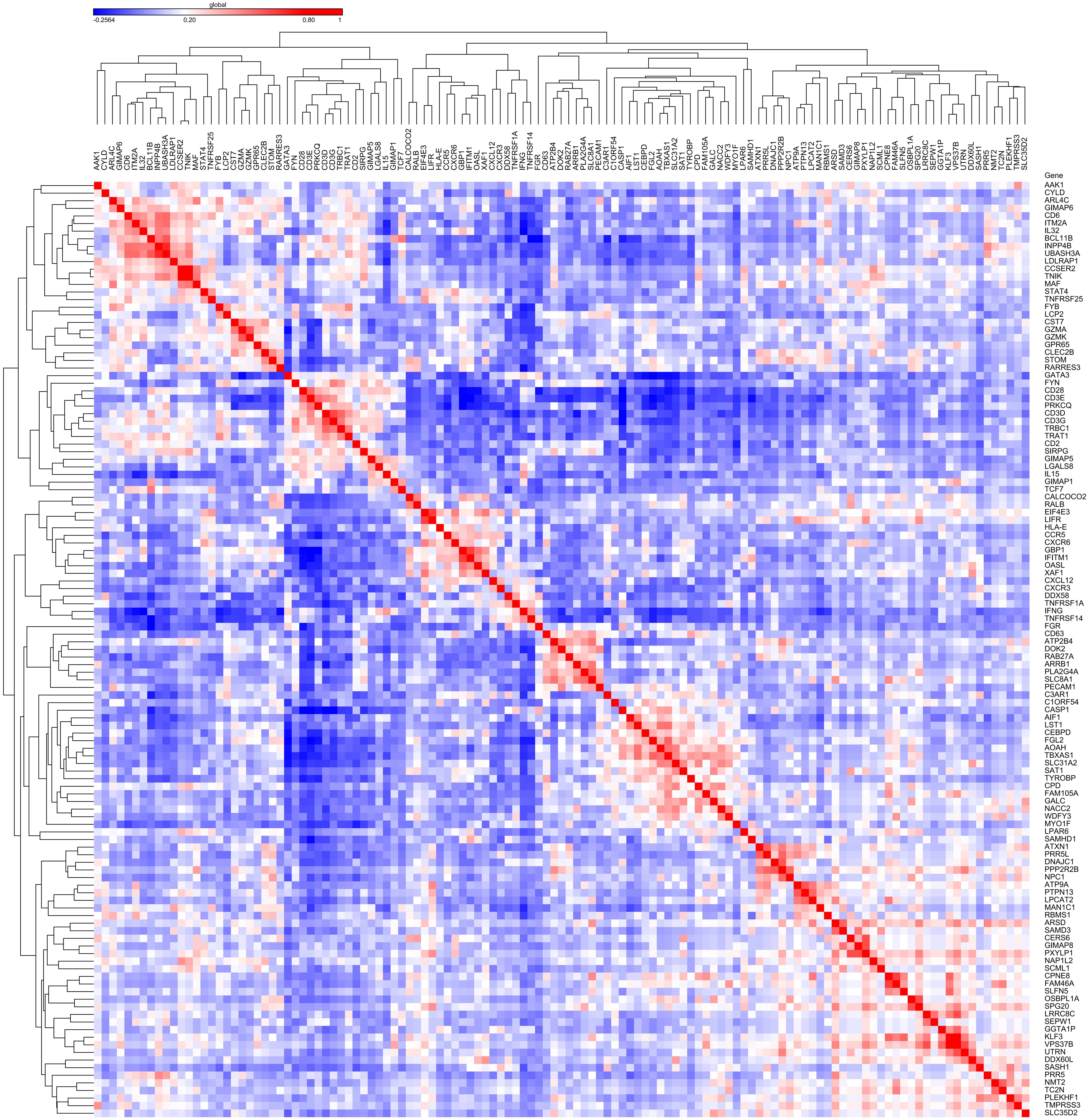
global
-0.2564
0.20
0.80
1
Gene
AAK1
CYLD
ARL4C
GIMAP6
CD6
ITM2A
IL32
BCL11B
INPP4B
UBASH3A
LDLRAP1
CCSER2
TNIK
MAF
STAT4
TNFRSF25
FYB
LCP2
CST7
GZMA
GZMK
GPR65
CLEC2B
STOM
RARRES3
GATA3
FYN
CD28
CD3E
PRKCQ
CD3D
CD3G
TRBC1
TRAT1
CD2
SIRPG
GIMAP5
LGALS8
IL15
GIMAP1
TCF7
CALCOCO2
RALB
EIF4E3
LIFR
HLA-E
CCR5
CXCR6
GBP1
IFITM1
OASL
XAF1
CXCL12
CXCR3
DDX58
TNFRSF1A
IFNG
TNFRSF14
FGR
CD63
ATP2B4
DOK2
RAB27A
ARRB1
PLA2G4A
SLC8A1
PECAM1
C3AR1
C1ORF54
CASP1
AIF1
LST1
CEBPD
FGL2
AOAH
TBXAS1
SLC31A2
SAT1
TYROBP
CPD
FAM105A
GALC
NACC2
WDFY3
MYO1F
LPAR6
SAMHD1
ATXN1
PRR5L
DNAJC1
PPP2R2B
NPC1
ATP9A
PTPN13
LPCAT2
MAN1C1
RBMS1
ARSD
SAMD3
CERS6
GIMAP8
PXYLP1
NAP1L2
SCML1
CPNE8
FAM46A
SLFN5
OSBPL1A
SPG20
LRRC8C
SEPW1
GGTA1P
KLF3
VPS37B
UTRN
DDX60L
SASH1
PRR5
NMT2
TC2N
PLEKHF1
TMPRSS3
SLC35D2
