# Supplementary material for: Gene expression meta-analysis reveals immune response convergence on the IFNγ-STAT1-IRF1 axis and adaptive immune resistance mechanisms in lymphoma
Source: Genome Med. 2015 Sep 11;7(1):96. doi: 10.1186/s13073-015-0218-3 (PMC4566848; doi:10.1186/s13073-015-0218-3)
Supplement: Additional file 11: Figure S7. — Relates to Fig. 4. Genes most consistently associated with COO-unclassified DLBCL are related to a polarized immune response. As in Fig. 4, the two principal branches of the gene-centred hierarchical clustering tree of the COO-unclassified meta-profile are illustrated on the left. Colour-coding above identifies: red cluster 1, corresponding to the T-cell cluster; black cluster 2, IFN and monocyte/immune NOS (not otherwise specified). On the right the relative rank of differentially expressed genes contributing to the COO-unclassified meta-profile is plotted using the median normalized fold change for gene ranking; the x-axis indicates differential expression rank in the comparison COO-unclassified versus ABC-DLBCL; the y-axis indicates differential expression rank in the comparison COO-unclassified versus GCB-DLBCL. Cluster membership is superimposed on the scatter plot of differential expression rank according to the colour coding shown (red cluster 1, black cluster 2). The 18 genes most consistently separating COO-unclassified DLBCL from either ABC- or GCB-DLBCL are illustrated below with cluster membership and mean differential expression rank shown. (PDF 275 kb) [file 13073_2015_218_MOESM11_ESM.pdf]

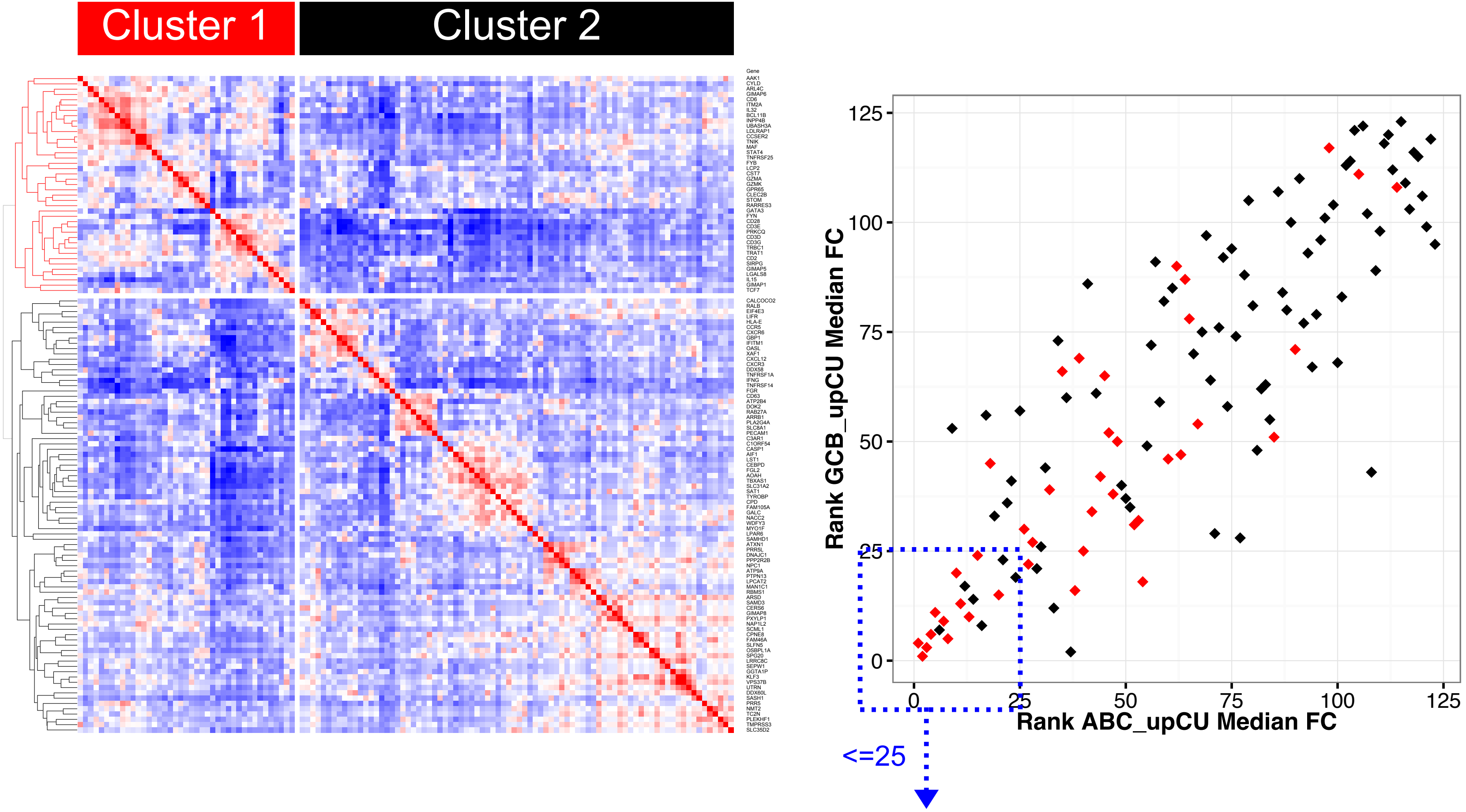

Gene	TRAT1	CD3D	RARRES3	GZMK	ITM2A	TC2N	CD2	CD3G	GIMAP6	IFNG	GZMA	CCR5	FGL2	CLEC2B	UBASH3A	TRBC1	CXCR6	C3AR1
Cluster	1	1	1	1	1	2	1	1	1	2	1	2	2	1	1	1	2	2
MeanRank	2	3	3	5	7	7	8	8	12	12	12	14	15	15	18	20	22	22
