# Supplementary material for: Gene expression meta-analysis reveals immune response convergence on the IFNγ-STAT1-IRF1 axis and adaptive immune resistance mechanisms in lymphoma
Source: Genome Med. 2015 Sep 11;7(1):96. doi: 10.1186/s13073-015-0218-3 (PMC4566848; doi:10.1186/s13073-015-0218-3)
Supplement: Additional file 13: Figure S8. — Relates to Fig. 5. Ranking by the 16-gene polarized immune response score demonstrates common occurrence of a polarized T-cell response across DLBCL from all data sets. Shown are all DLBCL data sets used with cases ranked by the 16-gene polarized immune response score. The data set number is shown above each heat map, followed by three bars: top bar COO class (yellow ABC, blue GCB, green unclassified); middle bar class confidence assigned during classification (blue low confidence to red high confidence); bottom bar polarized score (blue low polarized immune response score to red high polarized immune response score). These are followed by case-by-case gene expression values (illustrated as z scores) which are broken down into components identified by coloured bars on the right of each heatmap. The contributing genes are shown in the grey expanded box to the right of the figure with corresponding colour code: yellow bar ABC COO-classifier genes; blue bar GCB COO-classifier genes; green bar polarized immune response score; black bar extended COO-unclassified meta-profile and immune response genes. (PDF 1073 kb) [file 13073_2015_218_MOESM13_ESM.pdf]

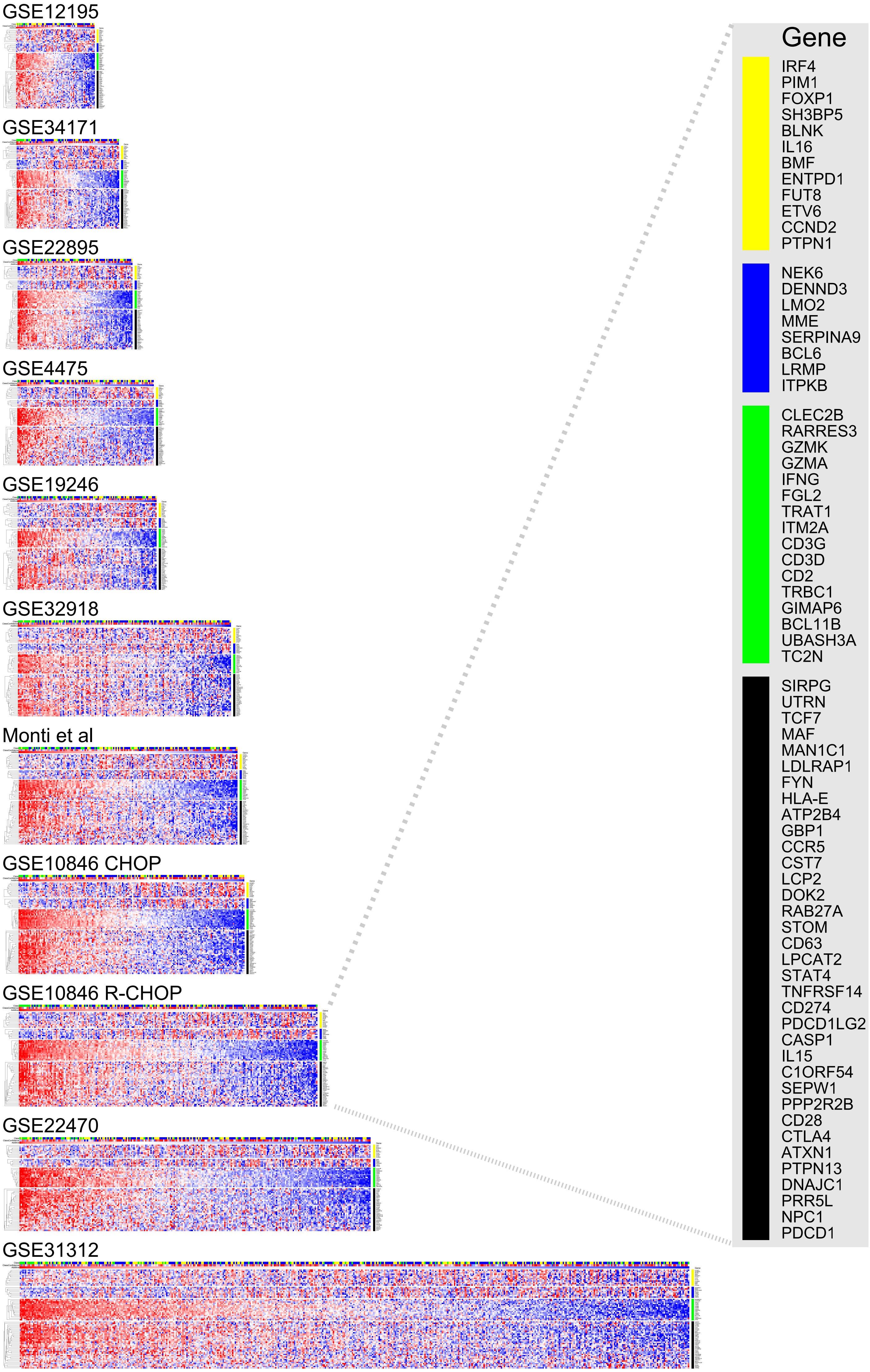
GSE12195
GSE34171
GSE22895
GSE4475
GSE19246
GSE32918
Monti et al
GSE10846 CHOP
GSE10846 R-CHOP
GSE22470
GSE31312
Gene
IRF4
PIM1
FOXP1
SH3BP5
BLNK
IL16
BMF
ENTPD1
FUT8
ETV6
CCND2
PTPN1
NEK6
DENND3
LMO2
MME
SERPINA9
BCL6
LRMP
ITPKB
CLEC2B
RARRES3
GZMK
GZMA
IFNG
FGL2
TRAT1
ITM2A
CD3G
CD3D
CD2
TRBC1
GIMAP6
BCL11B
UBASH3A
TC2N
SIRPG
UTRN
TCF7
MAF
MAN1C1
LDLRAP1
FYN
HLA-E
ATP2B4
GBP1
CCR5
CST7
LCP2
DOK2
RAB27A
STOM
CD63
LPCAT2
STAT4
TNFRSF14
CD274
PDCD1LG2
CASP1
IL15
C1ORF54
SEPW1
PPP2R2B
CD28
CTLA4
ATXN1
PTPN13
DNAJC1
PRR5L
NPC1
PDCD1
