# Supplementary material for: Gene expression meta-analysis reveals immune response convergence on the IFNγ-STAT1-IRF1 axis and adaptive immune resistance mechanisms in lymphoma
Source: Genome Med. 2015 Sep 11;7(1):96. doi: 10.1186/s13073-015-0218-3 (PMC4566848; doi:10.1186/s13073-015-0218-3)
Supplement: Additional file 15: Figure S10. — Clustering of COO classes and PMBL signature genes. Shown are all DLBCL data sets used, hierarchically clustered by all genes shown, including the 23-gene PMBL signature, and constrained by COO class. The data set number is shown above each heatmap, followed by three bars: top bar COO class (yellow ABC, blue GCB, green unclassified); middle bar class confidence assigned during classification (blue low confidence to red high confidence); bottom bar polarized score (blue low polarized immune response score to red high polarized immune response score). These are followed by case-by-case gene expression values (illustrated as z scores), which are broken down into components identified by coloured bars on the right of each heatmap. The contributing genes are shown in the grey expanded box to the right of the figure with corresponding color code: yellow bar ABC COO-classifier genes; blue bar GCB COO-classifier genes; green bar polarized immune response score; black bar PMBL 23-gene signature. (PDF 1002 kb) [file 13073_2015_218_MOESM15_ESM.pdf]

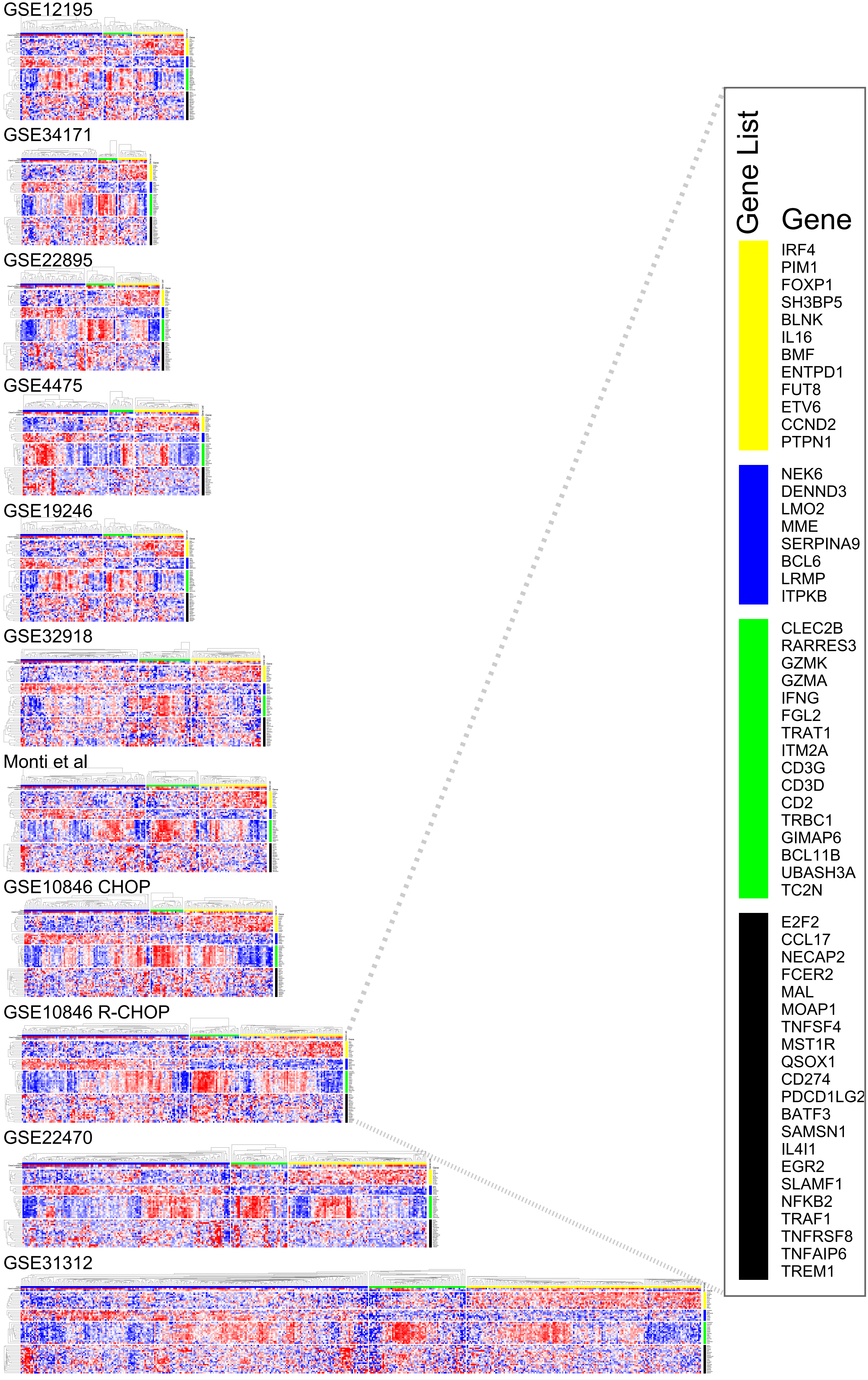
GSE12195
GSE34171
GSE22895
GSE4475
GSE19246
GSE32918
Monti et al
GSE10846 CHOP
GSE10846 R-CHOP
GSE22470
GSE31312
Gene List
Gene
IRF4
PIM1
FOXP1
SH3BP5
BLNK
IL16
BMF
ENTPD1
FUT8
ETV6
CCND2
PTPN1
NEK6
DENND3
LMO2
MME
SERPINA9
BCL6
LRMP
ITPKB
CLEC2B
RARRES3
GZMK
GZMA
IFNG
FGL2
TRAT1
ITM2A
CD3G
CD3D
CD2
TRBC1
GIMAP6
BCL11B
UBASH3A
TC2N
E2F2
CCL17
NECAP2
FCER2
MAL
MOAP1
TNFSF4
MST1R
QSOX1
CD274
PDCD1LG2
BATF3
SAMSN1
IL4I1
EGR2
SLAMF1
NFKB2
TRAF1
TNFRSF8
TNFAIP6
TREM1
