# Supplementary material for: Gene expression meta-analysis reveals immune response convergence on the IFNγ-STAT1-IRF1 axis and adaptive immune resistance mechanisms in lymphoma
Source: Genome Med. 2015 Sep 11;7(1):96. doi: 10.1186/s13073-015-0218-3 (PMC4566848; doi:10.1186/s13073-015-0218-3)

TRAT1

Signature Correlation

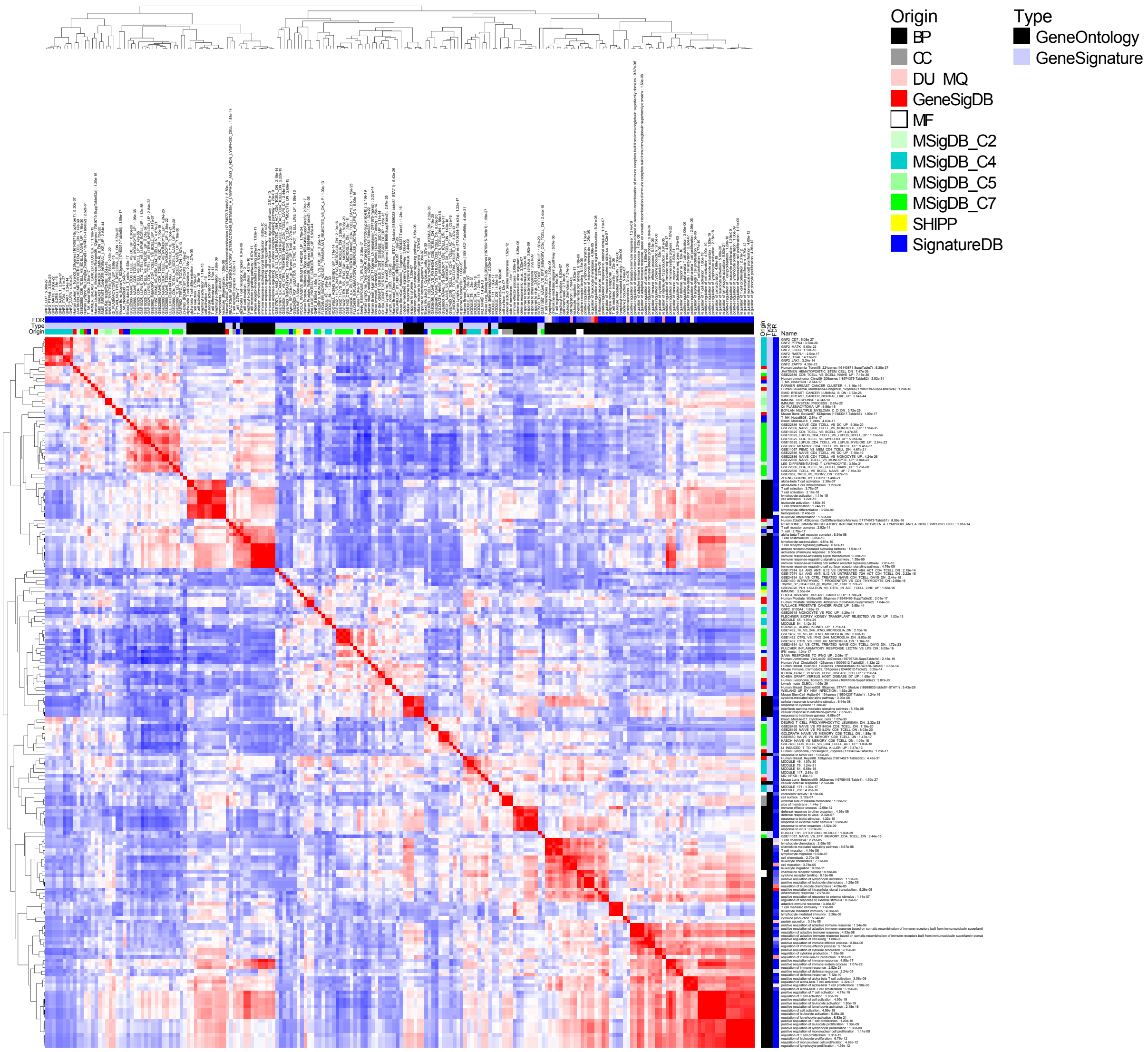

Gene Correlation

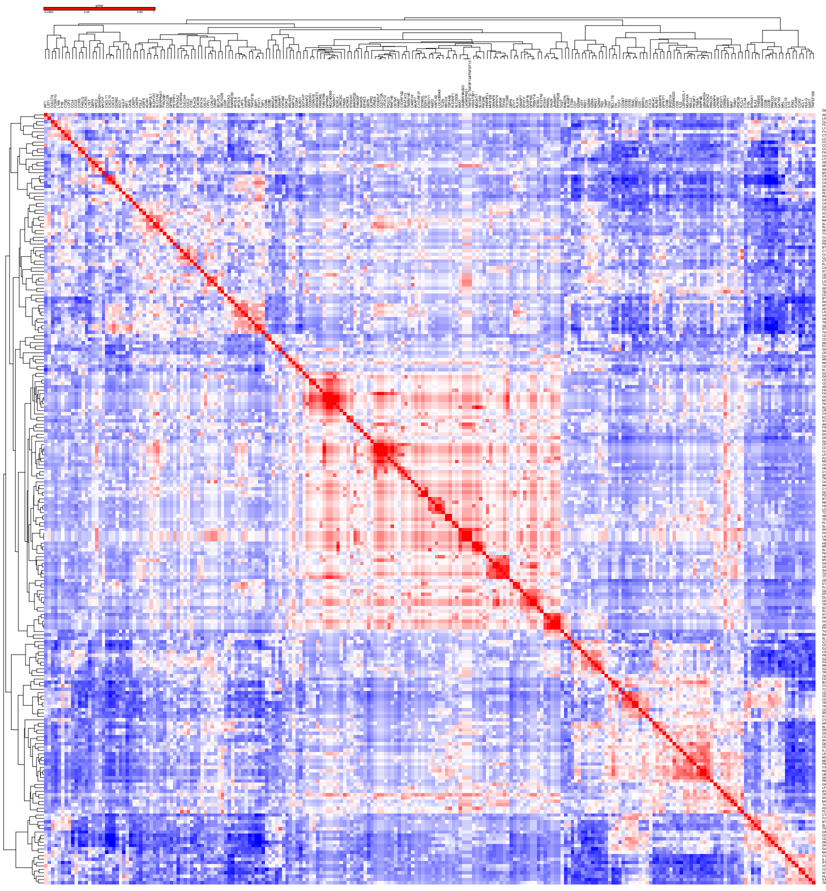

Supplement: Additional file 18: Figure S12. — Relates to Fig. 8b. Integrated gene signature and ontology enrichments for TRAT1 focus gene analysis. Upper panel: the hierarchical clustering of enriched gene signature and GO terms related to genes correlating with TRAT1 in focus gene analysis. Correlations are illustrated in the heatmap on a blue (least) to red (most) scale as indicated at the top of the figure. Along the edges of the heatmap the signature terms are provided (and correspond to terms listed in Additional file 17). The FDR-corrected p value for enrichment of the signature is illustrated as a bar on either side of the heatmap, along with an indication of the type of term (signature versus ontology) and the origin of the terms as indicated in the figure. Lower panel: the hierarchical clustering of genes correlating with TRAT1 in focus gene analysis. Genes shown are clustered according to the correlation of enriched signature/GO term membership. Correlations are illustrated in the heatmap on a blue (least) to red (most) scale as indicated at the top of the figure. Along the edges of the heatmap official gene symbols are provided. (PDF 1104 kb) [file 13073_2015_218_MOESM18_ESM.pdf]
